# Soluble prefusion-closed HIV-envelope trimers with glycan-covered bases

**DOI:** 10.1016/j.isci.2023.107403

**Published:** 2023-07-15

**Authors:** Adam S. Olia, Cheng Cheng, Tongqing Zhou, Andrea Biju, Darcy R. Harris, Anita Changela, Hongying Duan, Vera B. Ivleva, Wing-Pui Kong, Li Ou, Reda Rawi, Yaroslav Tsybovsky, David J. Van Wazer, Angela R. Corrigan, Christopher A. Gonelli, Myungjin Lee, Krisha McKee, Sandeep Narpala, Sijy O’Dell, Danealle K. Parchment, Erik-Stephane D. Stancofski, Tyler Stephens, Ivy Tan, I-Ting Teng, Shuishu Wang, Qing Wei, Yongping Yang, Zhengrong Yang, Baoshan Zhang, VRC Production Program, Jan Novak, Matthew B. Renfrow, Nicole A. Doria-Rose, Richard A. Koup, Adrian B. McDermott, Jason G. Gall, Q. Paula Lei, John R. Mascola, Peter D. Kwong

**Affiliations:** 1 Vaccine Research Center, National Institutes of Health, Bethesda, MD 20892, USA; 2 Electron Microscopy Laboratory, Cancer Research Technology Program, Leidos Biomedical Research, Inc, Frederick National Laboratory for Cancer Research, Frederick, MD 21702, USA; 3 Department of Microbiology, University of Alabama at Birmingham, Birmingham, AL, USA; 4 Department of Biochemistry and Molecular Genetics, University of Alabama at Birmingham, Birmingham, AL, USA

**Keywords:** Molecular structure, Virology

## Abstract

Soluble HIV-1-envelope (Env) trimers elicit immune responses that target their solvent-exposed protein bases, the result of removing these trimers from their native membrane-bound context. To assess whether glycosylation could limit these base responses, we introduced sequons encoding potential *N*-linked glycosylation sites (PNGSs) into base-proximal regions. Expression and antigenic analyses indicated trimers bearing six-introduced PNGSs to have reduced base recognition. Cryo-EM analysis revealed trimers with introduced PNGSs to be prone to disassembly and introduced PNGS to be disordered. Protein-base and glycan-base trimers induced reciprocally symmetric ELISA responses, in which only a small fraction of the antibody response to glycan-base trimers recognized protein-base trimers and vice versa. EM polyclonal epitope mapping revealed glycan-base trimers –even those that were stable biochemically– to elicit antibodies that recognized disassembled trimers. Introduced glycans can thus mask the protein base but their introduction may yield neo-epitopes that dominate the immune response.

## Highlights

• Designed soluble Env trimers with introduced *N*-linked glycans to mask base
• Glycan addition substantially reduced recognition by base-directed antibodies
• Immunization with protein- or glycan-base Env induced reciprocally symmetric ELISAs
• Antibodies elicited by glycan-base trimers disassembled nominally stable trimers

## Graphical abstract

**Figure:**
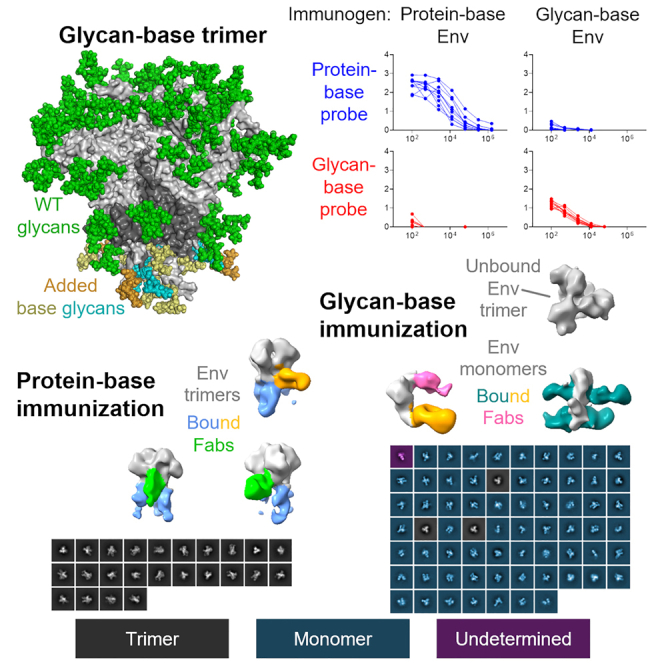

## Introduction

The HIV-1 envelope (Env) trimer, comprising three gp120 subunits and three gp41-transmembrane subunits, uses multiple strategies to evade the elicitation of neutralizing antibodies. It changes shape from closed conformations to open conformations^1^ and disassembles into highly immunogenic subunits,^2^^,^^3^ which elicit antibodies incapable of neutralizing the tier-2 resistant isolates that typify HIV-1 transmission. Thus, HIV-1 infected individuals are rapidly antibody positive, but elicited antibodies are generally non-neutralizing. Indeed, broadly neutralizing antibodies are elicited in only a minority of infected individuals and only after years of infection and high viremia.^4^

The introduction of soluble Env trimers, stabilized in the prefusion-closed conformation, by artificial disulfides, helix-disrupting prolines, and other often structure-based alterations,^5^^,^^6^^,^^7^^,^^8^^,^^9^^,^^10^ fixes both conformational and disassembly issues, and these prefusion-closed soluble trimers could elicit autologous neutralization against Tier-2 neutralization strains.^11^ Analysis of the elicited response in mice and non-human primates, however, indicated a substantial portion of the response elicited by these soluble trimers to be directed to the exposed trimer base.^12^^,^^13^^,^^14^^,^^15^^,^^16^ While the exposed base comprises less than 10% of the trimer surface, the VRC 018 clinical study with the stabilized soluble trimer, BG505 DS-SOSIP, revealed >90% of the immune response to be directed to its protein base.^17^

How to mask the base so that it is less immunogenic? We and others have previously shown that adding *N*-linked glycans to specific sites on immunogens could alter the immune response, focusing the response to other epitopes and increasing the elicitation of B cells encoding desired responses.^18^^,^^19^ Here, we test the impact of adding *N*-linked glycans to the exposed trimer base. To soluble trimers stabilized in the prefusion-closed conformation, we added up to three sequons encoding potential *N-*linked glycosylation sites (PNGSs) to each of the sequence segments comprising the exposed protein base. We tested 16 variants of each prefusion-stabilized trimer, selecting 4 variants for expression, and then a single lead variant with six added PNGSs for characterization of glycosylation, for appropriate antigenicity, of structure by electron microscopy, and of immunogenicity in mice and guinea pigs. Overall, the results indicate that the base glycans did indeed alter the immune response, but their introduction generated neo-epitopes that dominated the elicited response.

## Results

### Design and selection of glycan-base Env trimers

We chose two prefusion stabilized trimers as initial templates, the aforementioned BG505 DS-SOSIP^5^ as well as a consensus clade C trimer stabilized by multiple substitutions (ConC),^20^ which we have manufactured for clinical assessment.^21^ Further, we added multiple prefusion stabilizing mutations to the BG505-DS-SOSIP template, including repair-and stabilized-based stabilizing mutations,^9^ 3Mut,^22^ and 2G mutations,^7^ and to the ConC trimer,^9^ we further stabilized by adding the DS substitution^5^ and altering the fusion peptide sequence by replacing an isoleucine with a leucine.^22^

**Figure 1:**
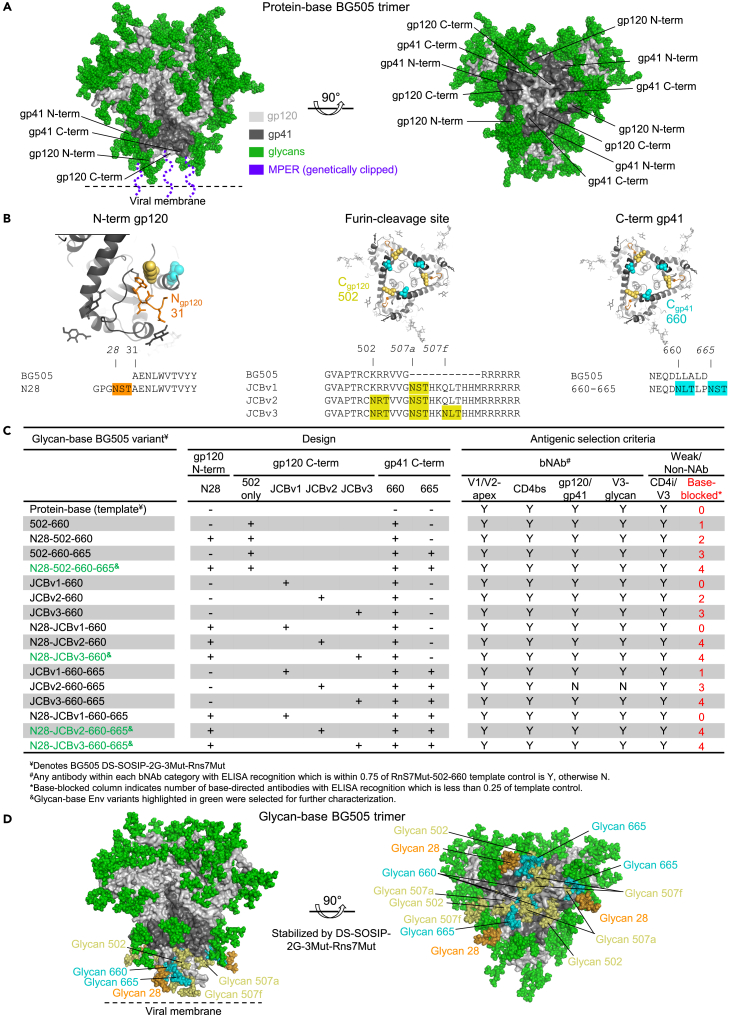
Design and selection of glycan base-covered Env trimers (A) BG505 DS-SOSIP Env trimer model highlighting sequence segments that comprise the exposed protein base. (B) Close-up view and sequence information showing location of N-linked glycans introduced in each sequence segment. Italicized numbers denote sequence insertions, and “BG505” refers to protein-base BG505 trimer. (C) Matrix of 16 Env variants and antigenic criteria used for selection. The first half of the table describes the glycan sequons that were added to each construct with + or – notation. The second half of the table summarizes the antibody binding of each construct, denoted with Yes or No binding, or in red with the number of base-directed antibodies blocked from binding. Constructs in green were selected for further characterization. (D) Glycan-base BG505 trimer, modeled with six-introduced N-linked glycans. See also Table S1.

The exposed Env-protein base on the template trimers was made up of three different sequence segments: the N-terminus of the gp120 subunit, the furin-cleavage site connecting the C-terminus of gp120 and the N-terminus of gp41, and the C terminus of gp41, where this subunit was genetically clipped from the membrane (Figure 1A). We introduced a PNGS at residue 28 at the N terminus of gp120, one or two PNGSs to the C terminus of gp41, and one to three PNGSs in the linker preceding the furin cleavage site at the gp120-gp41 juncture. The PNGSs at the furin site were added as part of a sequence, which we termed the “JCB-cleavage sequence”. This sequence derives from an influenza haemagglutinin variant,^23^ and we found its insertion upstream of the “RRRRRR” cleavage loop to result in more efficient cleavage, as well as enabling multiple PNGS additions (Figure 1B).

We synthesized constructs encoding each of the 16 glycan-base variants for both BG505 and ConC trimers. We used these to transfect HEK293-cells in a 96-well format, producing supernatants containing the glycan-variants, which we assessed antigenically for recognition by broadly neutralizing antibodies, for recognition by antibodies that bind open trimer conformations (F105, 447-52D, 17b, and 17b in the presence of CD4), and for recognition by five protein-base-directed antibodies (1E6, 5H3, 3H2, 989, and RM20A3) (Figure 1C; Table S1). In general, the antigenic properties of the glycosylated trimers were similar, with the main difference being the number of antibodies against the base that displayed reduced binding. Overall, trimers with 4–6 introduced PNGSs appeared to show the greatest reduction in base-directed antibody recognition, and we modeled these (Figure 1D) and selected four for further characterization.

### Physical properties of glycan-base Env trimers

**Figure 2:**
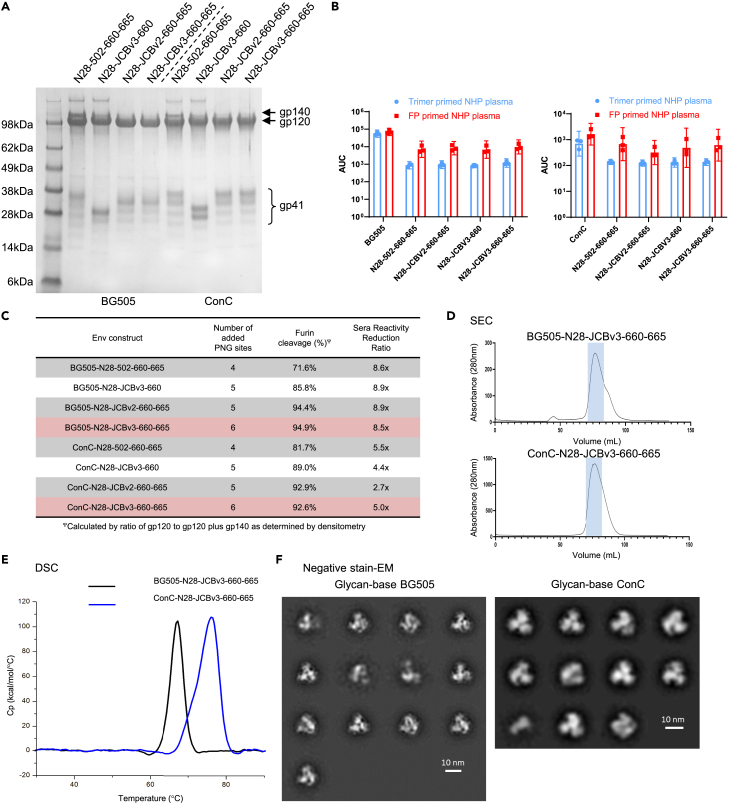
Physical properties of glycan base-covered Env trimers (A) SDS-PAGE of affinity purified glycan-base constructs from BG505 (left panel), and ConC (right panel). (B) ELISA response of sera from trimer-only or fusion peptide primed NHP immunizations assayed against selected glycan-base candidates. (C) Table showing the number of additional protein N-linked glycosylation (PNG) sites for each construct along with the percentage of furin cleavage, as determined by densitometric of the Coomassie gel in panel (A). Constructs highlighted in red were selected as the top candidates to move on for further characterization. (D) Size exclusion chromatography profiles of the two selected constructs with blue shading showing trimer containing fractions. (E) Differential scanning calorimetry scans of the two constructs, with calculated melting temperatures of 67.3°C and 76.5°C for the BG505 and ConC versions respectively. (F) Negative stain EM 2D class averages of BG505 and ConC glycan-base constructs. See also Figure S1.

The four selected constructs in both the BG505 and ConC background strains were expressed at larger scale, to allow for more thorough antigenic and biophysical characterizations. As a first pass of screening, the efficiency of furin cleavage was analyzed by comparing the intensity of the gp120 band on a Coomassie stained SDS-PAGE to that of the gp140 band (Figure 2A). Greater than 80% cleavage was observed for all constructs; for both BG505 and ConC, the most efficient cleavage was observed with constructs that include N28, JCBv2 or JCBv3, and 600–665 modifications (Figure 2C).

To test for the ability of the glycosylated bases to block immunoreactivity, the constructs for both BG505 and ConC were tested against sera from NHPs immunized with either the respective protein-base trimer alone or primed with fusion peptide and then boosted with protein-base trimer. As trimer alone sera primarily target the base of the HIV Env protein,^13^ and the fusion peptide primed sera are far less base-targeting, a reduction in the ratio of this reactivity would indicate successful blocking of this surface. All BG505 constructs, performed similarly, with an approximately 9-fold reduction in binding between the two sera. This ratio was more variable for the ConC constructs, ranging from an approximate 3- to 5-fold reduction in binding (Figures 2B and 2C). Constructs with the most added PNGSs, N28-JCBv3-660-665, for both BG505 and ConC, were selected for further analysis as they were the best cleaved, and in the case of ConC provided the highest differential in sera binding.

Both constructs of N28-JCBv3-660-665 for BG505 and ConC (hereafter referred to as BG505 glycan-base trimer and ConC glycan-base trimer) migrated through a size exclusion column as expected with the primary peak representing the trimeric fraction (Figure 2D). Moreover, differential scanning calorimetry (DSC) showed these two glycan-base trimers to be well-folded, stable proteins with T_m_’s of 67.3°C and 76.5°C for the BG505 and ConC variants, respectively (Figures 2E and S1). This stability is slightly reduced for BG505 compared to its protein-base counterpart, but the stability is increased for the ConC glycan-base constructs, likely owing to the additional DS stabilization added to the glycan variants. Finally, the trimeric nature of these proteins was confirmed by negative-stain electron microscopy (NS-EM), wherein both constructs appeared with the characteristic shape of HIV Env constructs, with a minimum of monomers or other misfolded particles seen (Figure 2F).

### Glycosylation, CD4 affinity, and antigenic profiles of BG505 and ConC glycan-base trimers

**Figure 3:**
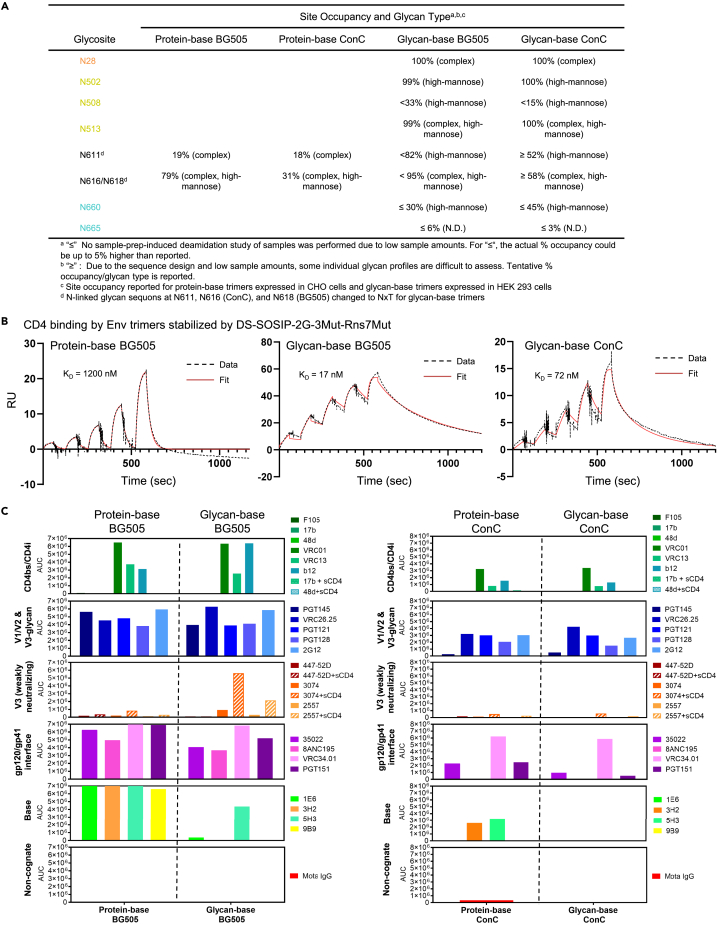
Glycosylation and antigenic profiles of BG505 and ConC base-covered trimers (A) Mass spectrometry glycosylation analysis of protein-base and glycan-base constructs of BG505 and ConC. (B) Surface plasmon resonance analysis of soluble CD4 binding to glycan-base BG505 and ConC, as compared to protein-base BG505. (C) MSD antibody binding analysis of glycan-base BG505 and ConC as compared to their protein-base counterparts, all stabilized by DS-SOSIP-2G-3Mut-RnS7Mut. See also Figures S2 and S3.

Glycosylation profiles and glycan occupancy of the six engineered PNGSs and other base glycans in gp41 were assessed for BG505 and ConC glycan-base trimer variants using LC-MS/MS peptide mapping methods (Figure 3A).^24^ The majority of base glycans were predominantly high-mannose type, except for the N28 glycosite which contained primarily complex-type glycan. Aside from the N508 glycan, all other engineered PNGSs within gp120 were nearly fully occupied (99–100%). In contrast, the engineered base glycans in gp41 displayed decreased occupancy, with the N660 glycan showing <45% occupancy and the C-terminal N665 glycan <6% occupancy. Native base glycans at N611 and N616 (ConC) or N618 (BG505) were found to have increased glycan occupancy in the glycan-base trimers compared to their protein-base counterparts, likely from the use of NxT sequons to optimize glycan occupancy.^25^ Interestingly, the glycan type for the N611 and N616/N618 sites switched from complex-type in the protein-base trimers to high-mannose type in the glycan-base trimers (Figure 3A), likely a consequence of increased glycosylation density around the base reducing glycan processing. Thus, the added base glycans appear to alter the processing of glycans throughout gp41. Interestingly, the glycosylation in gp120 was largely unchanged (Figure S2). At almost all sites in gp120, the difference in glycosylation occupancy between protein-base or glycan-base BG505 or ConC was less than 5%. The primary exceptions were at N182 in BG505 and N156 in ConC, which in both cases show a moderate decrease (∼15%) in occupancy in the glycan-base versions of the trimer.

To characterize further the glycan-base trimers, we assessed their binding affinities to a soluble construct of CD4 containing the first two domains (sCD4) by surface plasmon resonance (Figure 3B). Current data indicate CD4 affinity to be inversely related to the stability of the prefusion closed state of the Env trimer to which CD4 needs to bind and induce conformational transition to the open state for entry. For example, the CD4 affinity for the SOSIP-only stabilized trimer is about 1 nM, DS-SOSIP with an additional disulfide has an affinity of ∼10 nM, and further stabilized trimers show affinity for CD4 of 350–400 nM.^22^ The protein-base constructs analyzed here have additional RnS7mut stabilization,^26^ further decreasing their affinity to CD4 to the observed 1,200 nM. Glycan-base trimers follow the same overall trend, with the affinity of the glycan-base BG505 comparable to that previously reported for stabilized BG505.^22^

Antigenic profiles for the BG505 and ConC glycan-base trimers were evaluated in comparison to the protein-base counterparts using the Meso Scale Discovery (MSD) platform (Figures 3C and S3). Glycan-base and protein-base BG505 and ConC trimers exhibited comparable binding affinity to a panel of antibodies targeting the CD4-binding site (CD4bs), V1/V2-apex, V3-glycan, and gp120/gp41 interface; however, the glycan-base BG505 trimer (but not the ConC version) displayed increased binding to weakly neutralizing, V3-directed antibodies, 3074 and 2557. Notably, the glycan-base trimers showed reduced or no recognition by several base-directed antibodies, indicating the ability of the engineered glycans to mask the trimer base.

### Structure of ConC Env trimer with glycan-base trimers

**Figure 4:**
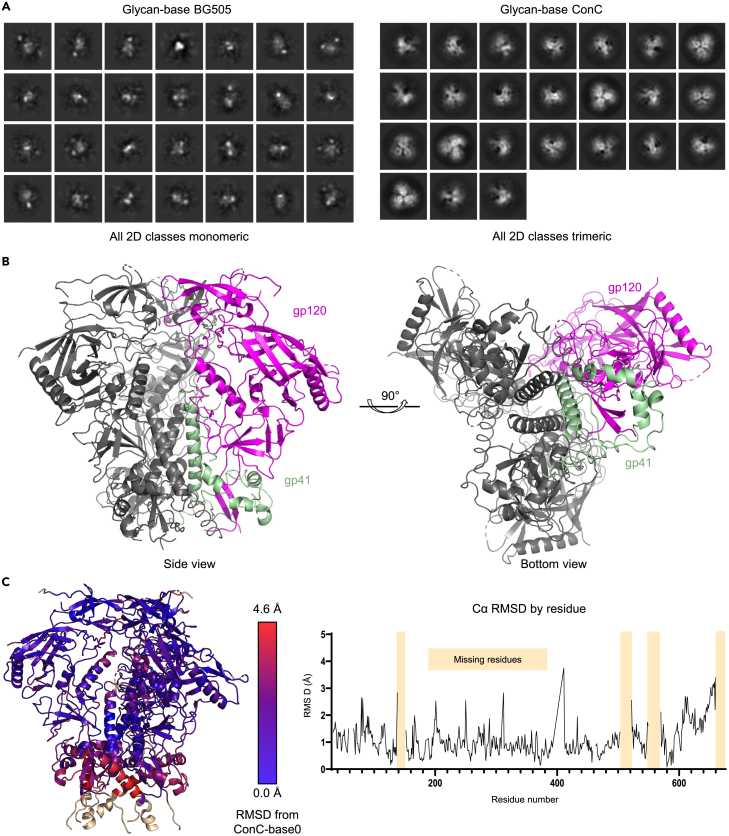
Cryo-EM of ConC Glycan-Base Trimer (A) Cryo-EM 2D class averages for BG505 and ConC glycan-base constructs. The classification for the BG505 glycan-base did not reveal any trimeric classes with all particles appearing as monomers, whereas ConC showed a nearly fully trimeric appearance. (B) Cryo-EM reconstruction of the ConC glycan-base trimer at 4.1 Å resolution. (C) Color-coded representation of RMSD of glycan-base ConC from protein-base ConC (PDB: 6CK9). Increasing RMSD are colored in red, and residues that could not be resolved in the glycan-base trimer reconstruction have been modeled and colored in wheat. See also Figures S4 and S5; Table S2.

To determine if there were changes in the structure of the HIV Env trimers resulting from the additional glycan motifs, we sought to determine the cryogenic electron microscopy (cryo-EM) structure of the glycan-base trimers. Surprisingly, even though the BG505 glycan-base trimer protein appeared nearly completely trimeric by negative-stain EM (Figure 2F), when imaged on frozen grids, the glycan-base BG505 protein appeared to disassemble, and few to no intact oligomers could be found (Figure 4A). Grids prepared with the glycan-base ConC trimer, however, yielded well-formed trimers.

We solved the cryo-EM structure of the glycan-base ConC trimer to 4.1 Å resolution (Figures 4B and S4; Table S2). Other than the additional DS mutation, the structure appeared generally indistinguishable from the published structure of the protein-base ConC (Figures 4C and S4).^9^ One difference observed in the structure, however, was an increase in the mobility in the base of the trimer (Figures 4C and S5). The base as a whole was shifted nearly 5 Å, and the C-terminal 10 residues were not resolved in the cryo-EM density. This unfortunately included the region where PNGSs were added, so we were not able to visualize the structure of the glycan additions. Moreover, the N-terminal addition (N28) and the addition proximal to the fusion peptide (JCBv3) were also absent from the electron density map, demonstrating that in all cases, the addition of the glycosylation sites, or the glycans themselves, increased the mobility of these regions.

### Murine immunogenicity reveals reciprocally symmetric responses

**Figure 5:**
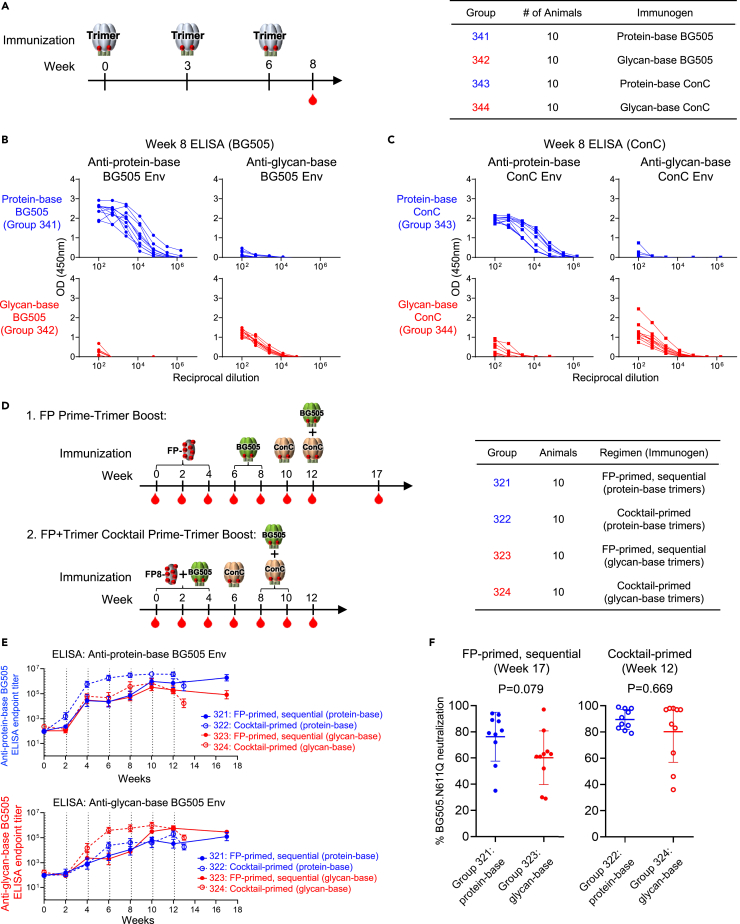
Trimer immunogenicity in mice reveals reciprocally symmetric responses (A) Immunization scheme and groups are shown for mice immunized with protein-base (blue) or glycan-base (red) variants of BG505 and ConC Env trimers. (B) Anti-trimer ELISA responses at week 8 for BG505 trimer-immunized groups and (C) ConC trimer-immunized groups. (D) Immunization schema and groups are shown for mice immunized with protein-base or glycan-base trimers using FP-primed, sequential or FP + trimer cocktail-primed vaccine regimens. (E) Longitudinal trimer-specific serum immunogenicity is shown for each group, as assessed by ELISA against protein-base BG505 trimer (top) or glycan-base BG505 trimer (bottom). Immunization timepoints are indicated by vertical dotted lines. (F) Percent neutralization against BG505.N611Q virus is shown after the final immunization using a 1:50 serum dilution for FP-primed, sequential groups (left) and cocktail-primed groups (right). Mean ± SD are shown. See also Figure S6 and Table S4.

To evaluate the immunogenicity of the glycan-base Env trimers, four groups of C57BL/6 mice (n = 10/group) were immunized three times at 3-week intervals with either protein-base or glycan-base trimers of BG505 and ConC (Figure 5A). Serum anti-trimer ELISA responses against either protein-base or glycan-base trimers were measured for each group at week 8, two weeks following the third trimer immunization (Figures 5B and 5C). ELISA titers elicited by each group were reciprocally symmetric, with only a small fraction of the antibodies elicited by glycan-base BG505 or ConC trimer able to recognize the corresponding protein-base trimer, and vice versa (Figures 5B, 5C, and S6). Similar to observations reported by others, no neutralizing activity (ID_50_) was detected against the autologous BG505 and ConC pseudoviruses for any of the mice at week 8 after the third trimer immunization, although sera from one animal immunized with the glycan-base ConC trimer (Group 344) did show neutralizing activity against the heterologous tier-1 MW965.26 strain (Figure S6).

Prior studies have shown that an epitope-focused vaccine approach based on priming with HIV-1 fusion peptide (FP)-carrier conjugates and boosting with prefusion-closed stabilized HIV-1 Env trimers can elicit cross-clade HIV-1 neutralizing responses in mice, guinea pigs and rhesus macaques.^27^^,^^28^^,^^29^^,^^30^ To assess the utility of glycan-base trimers as boosting reagents, the immunogenicity of the glycan-base trimers was evaluated in the context of two different FP-primed vaccine regimens (Figure 5D). Two groups of C57BL/6 mice (n = 10/group) were immunized following an FP-primed trimer-boosted sequential regimen whereby animals were primed three times every two weeks with FP8v1-rTTHC, an immunogen comprising the N-terminal eight residues of the most prevalent HIV-1 FP sequence (FP8v1) conjugated to a recombinant version of the tetanus toxoid heavy chain fragment C (rTTHC) as a carrier protein.^31^ After FP priming, animals were boosted sequentially with either the protein-base or glycan-base BG505 trimer two times, followed by a single immunization with the ConC trimer and finally once with a BG505 + ConC trimer cocktail. Another two groups of mice followed a “cocktail-primed” regimen whereby animals were primed three times every two weeks with a cocktail of FP8v1-rTTHC combined with either the protein-base or glycan-base variant of BG505 trimer. Following the FP + trimer cocktail prime, animals were boosted once with the ConC trimer variant and twice with a BG505+ConC trimer cocktail.

The longitudinal development of serum antibody responses to trimers was measured by ELISA against either the protein-base or glycan-base BG505 trimer (Figure 5E). In general, anti-trimer ELISA titers developed following the second FP or FP + trimer immunization and increased gradually throughout the trimer boosts for each group. For the FP + trimer cocktail-primed groups, ELISA titers were overall higher against the matching trimer immunogen during the course of immunization whereas FP-primed, sequential groups induced more similar responses against each trimer.

After the final immunization, sera were assessed at a 1:50 dilution for neutralizing activity against the BG505.N611Q viral variant, which lacks the N611 glycan resulting in enhanced sensitivity to FP-targeted neutralization.^27^ Neutralizing activity against the BG505.N611Q strain was detected for all groups after the last immunization, while the protein-base and glycan-base trimers induced similar neutralizing responses, with no statistically significant difference observed between groups for each vaccine regimen (Figure 5F).

### Glycan-base BG505 trimer elicits immune responses in Guinea pigs capable of disassembling trimer

**Figure 6:**
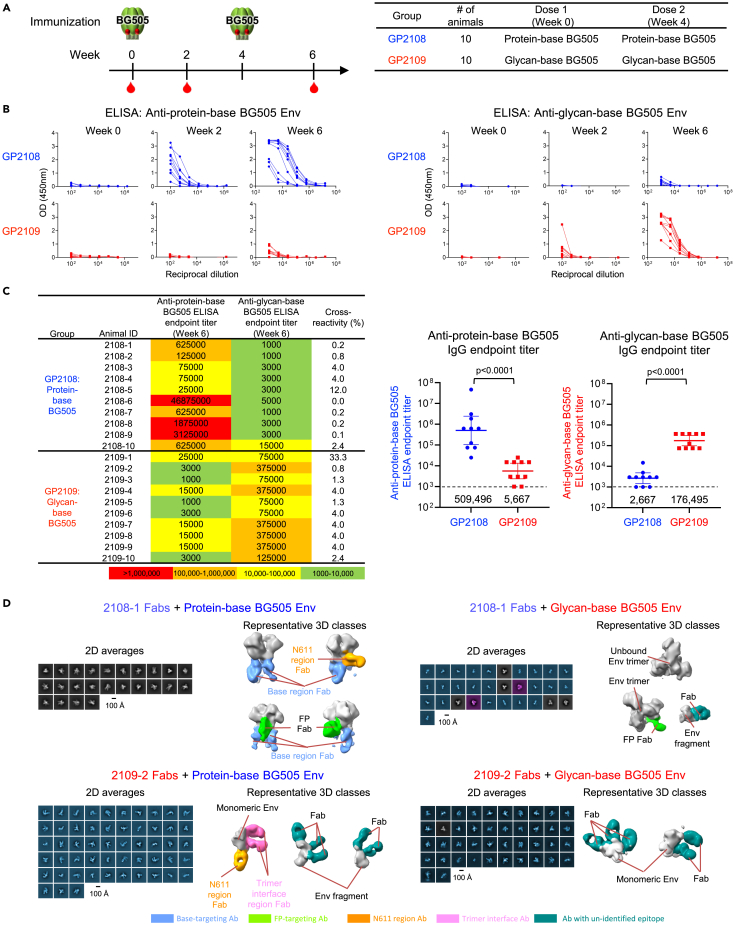
Glycan-base BG505 trimer elicit responses capable of dissembling trimer (A) Immunization scheme and groups are shown for guinea pigs immunized with protein-base (blue) or glycan-base (red) BG505 trimer variants. (B) Anti-trimer ELISA responses at weeks 0, 2 and 6 are shown for each group against protein-base BG505 Env (left) and glycan-base BG505 Env (right). (C) Week 6 ELISA endpoint titers using a starting dilution of 1:1000 and cross-reactivity (%) of responses are listed for each animal (left) and group comparison shown for week 6 titers against each trimer (right). (D) Electron microscopy of polyclonal epitope mapping (EMPEM) of antibodies elicited by immunization. Fabs were generate from whole plasma IgG and incubated with indicated HIV-1 Env. Negative stain EM were carried out using Env-Fab complexes purified by size exclusion chromatography, 2D averages were shown to the left of each panel, false colored in gray for trimeric particles, cyan for monomeric particles, and purple for undetermined. Representative 3D reconstructions were shown to the right with HIV-1 Env or Env fragments colored in gray, and Fabs colored by potential targeting sites. See also Tables S3 and S4.

To obtain a more comprehensive understanding of the immune responses elicited by the glycan-base Env trimers, we also immunized guinea pigs with either glycan-base or protein-base BG505 trimers. In the first set of experiments, animals (n = 10/group) were immunized twice, 4 weeks apart, with either the protein-base or glycan-base BG505 trimer, administered with Adjuplex adjuvant (Figure 6A). Anti-trimer ELISA responses against either protein-base or glycan-base BG505 trimer were measured for each group at 0, 2, and 6 weeks and showed an increase in responses after each immunization with the corresponding trimer immunogen (Figure 6B). Similar to the responses observed in mice, ELISA titers elicited by each group appeared reciprocally symmetric, with the response to either the glycan-base or protein-base BG505 trimer showing low cross-reactivity to the alternate trimer (Figures 6B and 6C).

Neutralization activity was assessed at week 6, two weeks following the second trimer immunization, using pseudoviruses produced with wild-type BG505, BG505.N611Q, and MW965.26 HIV-1 envelope, and the envelope of 7312A_V434M, an HIV-2 strain with enhanced sensitivity to CD4-induced antibodies (Tables S3 and S4). Animals immunized with the protein-base BG505 trimer elicited consistent neutralizing responses against the autologous wildtype BG505 (8 of 10 animals) and BG505.N611Q (10 of 10 animals) viruses, while only sporadic neutralizing activity against BG505 was observed for the glycan-base BG505 trimer-immunized group (2 of 10 animals). Neutralizing activity against the heterologous tier 1 MW965.26 strain was similar between the two groups and there was little response to the HIV-2 7312A_V434M virus.

To map the antibody responses elicited by protein-base and glycan-base BG505 trimers, we performed electron microscopy polyclonal epitope mapping (EMPEM)^32^ using sera collected at week 6, two weeks following the second trimer dose, from one animal in each group (Figure 6D). Antibodies elicited by immunization with the protein-base BG505 trimer were observed to target the base, N611, and FP regions of the trimer, consistent with other studies with SOSIP-stabilized Env trimers.^12^^,^^14^^,^^15^^,^^33^ While trimer disassembly has been previously reported,^34^ with the more highly stabilized protein-base Env used here, we did not observe this phenomena. However, in line with the propensity of the glycan-based BG505 Env trimers to dissociate, we observed vaccine-elicited antibodies from glycan-base trimer immunizations to recognize disassembled glycan-base trimers, as indicated by Fabs binding to Env monomers and fragments. Notably, representative 3D classes from samples made with antibodies elicited by the glycan-base trimers appeared to disassemble the nominally stable protein-base BG505 Env trimers.

### Glycan-base ConC trimer also elicits responses capable of recognizing disassembled trimers

**Figure 7:**
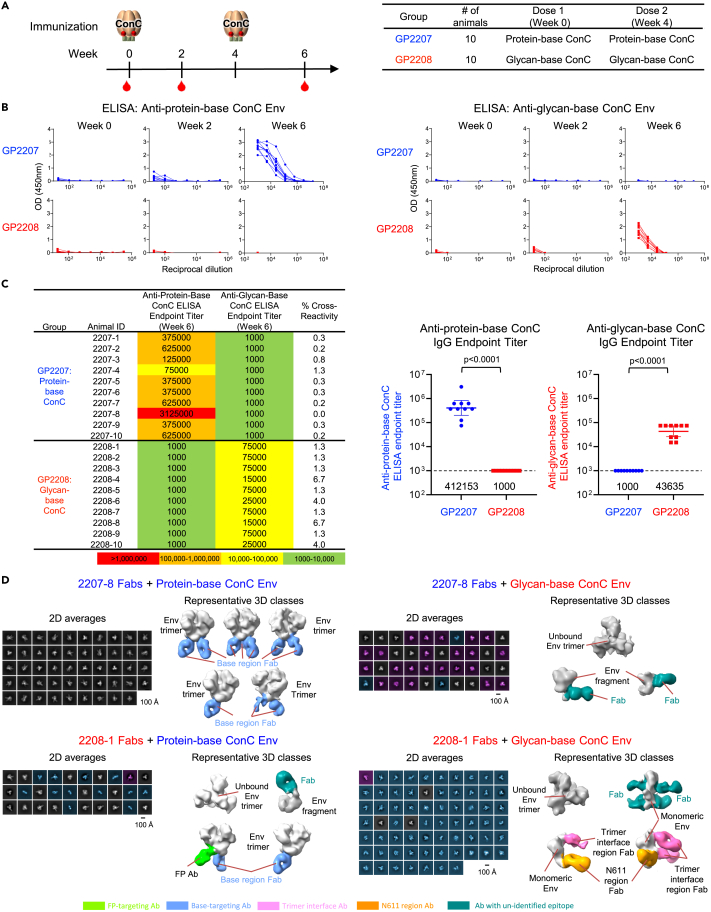
ConC trimer immunogenicity in guinea pigs reveals reciprocally symmetric responses (A) Immunization scheme and groups shown for guinea pigs immunized with protein-base (blue) or glycan-base (red) ConC Env trimers. (B) Anti-trimer ELISA responses at weeks 0, 2 and 6 are shown for each group against protein-base ConC Env (left) and glycan-base ConC Env (right). (C) Week 6 ELISA endpoint titers using a starting dilution of 1:1000 and cross-reactivity (%) of responses are listed for each animal (left) and group comparison shown for week 6 titers against each ConC trimer (right). (D) Electron microscopy of polyclonal epitope mapping (EMPEM) of antibodies elicited by immunization. Fabs were generate from whole plasma IgG and incubated with indicated HIV-1 Env. Negative stain EM were carried out using Env-Fab complexes purified by size exclusion chromatography, 2D averages were shown to the left of each panel, false colored in gray for trimeric particles, cyan for monomeric particles, and purple for undetermined. Representative 3D reconstructions were shown to the right with HIV-1 Env or Env fragments colored in gray, and Fabs colored by potential targeting sites. See also Tables S3 and S4.

Parallel immunogenicity studies were done in guinea pigs using protein-base and glycan-base ConC trimers. Two groups of animals (n = 10/group) were immunized twice, 4 weeks apart, with either the protein-base or glycan-base ConC trimer (Figure 7A). Anti-trimer ELISA responses followed similar trends to that observed with the BG505 trimers, showing reciprocal binding responses (Figure 7B) and low cross-reactivity to the alternate trimer (Figure 7C). No neutralizing activity (ID_50_) was detected against the wildtype or N611Q variant of ConC pseudovirus for any of the animals from either group at week 6 after the second trimer immunization, although sera from two animals immunized with the glycan-base ConC trimer showed neutralizing activity against the heterologous MW965.26 strain (Table S3).

EMPEM analysis was carried out with week 6 sera collected after the second trimer dose from one animal in each group (Figure 7D). As expected, we observed antibodies elicited by immunization with the protein-base ConC trimer to target the base and FP regions of the protein-base ConC trimer. However, despite the increased stability of the glycan-base ConC trimers, and no indication antigenically of their disassembling, in the case of antibodies elicited by immunization with the glycan-based ConC trimer, we observed few bound trimers, but instead predominantly observed bound fragments of disassembled ConC trimer. Vaccine-elicited antibodies from glycan-base ConC trimer immunizations were thus observed to bind base regions of the intact protein-base ConC trimer and predominantly recognized disassembled glycan-base ConC trimers.

## Discussion

To evade immune recognition, viruses utilize glycosylation to cover exposed antigenic protein surfaces. The HIV-1 Env is heavily glycosylated with *N*-linked glycans that shield potential neutralizing epitopes. Soluble Env trimer immunogens that have been removed by genetic cleavage from their native membrane-bound form, however, have exposed protein bases that become immunogenically dominant.^13^ In this study, we introduced *N*-linked glycans to cover the artificial protein base of soluble Env trimers, in an attempt to focus immune responses on the neutralizing epitopes. Antigenically, we observed that trimers with six-introduced PNGSs had reduced base responses, and immunogenically these trimers elicited reciprocally symmetric ELISA titers.

While the BG505 glycan-base trimer did show antigenic evidence of a greater propensity to open (e.g., increased binding to V3 antibodies in the presence of CD4), the ConC glycan-base trimers did not display signs of antigenically opening or disassembling and thus appeared biochemically stable *in vitro*. The ConC cryo-EM structure, however, did reveal greater mobility in the base region, and all of the introduced PNGSs were disordered in the electron density. Moreover, EMPEM of the elicited response showed clear evidence of elicited antibodies recognizing disassembled trimers, and these antibodies were capable of dissociating nominally stable protein-base versions of both BG505 and ConC (Figures 6 and 7). We note in this context that Turner and colleagues also observed base-directed antibodies to destabilize Env trimers.^34^

A possible explanation for the preferential elicitation of antibodies directed against monomers of the Env trimer comes from the recently published results which show that reduced germinal center recruitment can lead to antigen degradation.^35^ In the case of the soluble protein-base constructs, the most immunogenic region is the base of the trimer, with the remainder of the glycan-dense Env surface having extremely low immunogenicity. As such, once we removed the protein base of these trimer as a reactive site in the glycan-base constructs, the trimer no longer had a surface which the immune system could “latch onto”, leading to reduced germinal center recruitment – and greater degradation, exposing immunogenic sites not normally accessible, including the trimeric interface.

Despite the shortcomings of the current glycan-base trimers, the glycan-base ConC trimer may have utility as a boosting immunogen, as we show with mice primed using FP8-rTTHC (or cocktail of FP8-rTTHC and trimer) and boosted with glycan-base trimers elicited equivalent neutralizing responses to those boosted with protein-base trimers. Because the glycan-base trimers have reduced responses to base-targeting antibodies compared with the protein-base trimers, alternating boosts with glycan-base trimers and protein-base trimers may help to avoid the dominating base immunogenicity from the exposed protein base of the soluble trimers. The inclusion of either a peptide or protein-base trimer priming immunogen may provide an initial response which could then be used to enhance recruitment of the glycan-base trimer to the germinal center, potentially avoiding (or at least reducing) the degradation phenomena observed when using the glycan-base trimers alone.

Lastly, we note that one means to reduce or even eliminate the dominant immune response from the exposed protein base of the soluble Env trimers is to place these trimers in their native membrane-bound context, perhaps in the context of virus-like particles (VLPs) or as membrane-bound Env delivered by liposomes. Indeed, immunization with mRNA formatted VLPs presenting HIV-1 Env trimers does induce neutralizing antibodies in rhesus macaques, although elicited neutralizing titers are generally weak.^36^ It remains to be seen whether membrane-bound trimers or soluble trimers with antigenically masked bases will enable high titer neutralizing responses against HIV-1 to be elicited by vaccination.

### Limitations of the study

The main limitation of this study is the lack of a single clear mechanism for the elicitation of the antibodies which can disassemble nominally stabled trimers. While we show definitively that these antibodies are present in glycan-base immunized sera, it is unclear if the base modifications themselves induced this response, if the slightly reduced trimer stability of the glycan-base trimer was responsible, or if they arose from a more complex interplay between immune system and trimers, such as reduced capturing of the glycan-base trimers by germinal centers leading to their proteolysis and presentation of epitopes normally hidden within the trimer interface. While candidate trimers can be tested in animal models, a biochemical assay to predict this disassembly would be desirable but remains to be developed.

## Consortia

The VRC Production Program includes Nadia Amharref, Frank J. Arnold, Nathan Barefoot, Christopher Barry, Boonchai Boonyaratanakornkit, Elizabeth Carey, Ria Caringal, Kevin Carlton, Naga Chalamalsetty, Amy Lynch Chamberlain, Adam Charlton, Rajoshi Chaudhuri, Mingzhong Chen, Peifeng Chen, Yue Chen, Nicole Cibelli, Jonathan W. Cooper, Hussain Dahodwala, Marianna Fleischman, Julia C. Frederick, Haley Fuller, Mridul Ghosh, Isaac Godfroy, Deepika Gollapudi, Daniel Gowetski, Krishana Gulla, Joe Horwitz, Althaf Hussain, Tina Khin, Lisa Kueltzo, Gabriella Lagos, Yile Li, Slobodanka Manceva, Venkata Mangalampalli, Gabriel Moxey, Sarah O’Connell, Aakash Patel, Erwin Rosales-Zavala, Elizabeth Scheideman, Nicole A. Schneck, Zachary Schneiderman, Andrew Shaddeau, William Shadrick, Shamitha Shetty, Brad Tippett, Joseph Varriale, Alison Vinitsky, Hairong Wang, Xiangchun E. Wang, Calvin Webber, Sara Witter, Gengcheng J. Yang, Lu Yang, Yanhong Yang, and Yaqiu Zhang.

## STAR★Methods

### Key resources table

**Table:** 

REAGENT or RESOURCE	SOURCE	IDENTIFIER
**Antibodies**		

CAP256-VRC26.25	Doria-Rose et al.^37^	N/A
PGT145	Walker et al.^38^	N/A
PGDM1400	Sok et al.^39^	N/A
PGT151	Blattner et al.^40^	N/A
VRC34.01	Kong et al.^41^	N/A
35O22	Huang et al.^42^	N/A
PGT122	Walker et al.^38^	N/A
N6	Huang et al.^43^	N/A
3BNC117	Scheid et al.^44^	N/A
F105	Posner et al.^45^	N/A
447.52D	Sharon et al.^46^	N/A
17b	Thali et al.^47^	N/A
1E6	Cottrell et al.^14^	N/A
5H3	Cottrell et al.^14^	N/A
3H2	Cottrell et al.^14^	N/A
989	Cottrell et al.^14^	N/A
RM20A3	Cottrell et al.^14^	N/A

**Chemicals, peptides, and recombinant proteins**		

Pierce Protein A Agarose	ThermoFisher Scientific	20334
Turbo293 transfection reagent	SPEED BioSystem	PXX1002
AbBooster medium	ABI scientific	PB2668
FreeStyle 293 Expression Medium	ThermoFisher Scientific	12338018
Expi293 Expression Medium	ThermoFisher Scientific	A1435101
sCD4	Ryu et al.^48^	N/A
n-Dodecyl-β-D-Maltopyranoside	Anatrace	D310

**Deposited data**		

Glycan-base ConC Structure	PDB	8F7T
Glycan-base ConC Maps	EMDB	EMD-28910

**Experimental models: Cell lines**		

293F Freestyle cells	Thermo Fisher	K900001
Expi293F cells	Thermo Fisher	A14527

**Recombinant DNA**		

pVRC8400-glycan-base-trimers	This paper	N/A

**Software and algorithms**		

Phenix	Adams et al.^49^	https://sbgrid.org/software/
Coot	Emsley and Cowtan^50^	https://sbgrid.org/software/
Pymol	Schrödinger	https://pymol.org
EMAN2 software package	Tang et al.^51^	http://blake.bcm.edu/emanwiki/EMAN2
CryoSparc	Punjani et al.^52^	https://guide.cryosparc.com/
PRISM 7	GraphPad Software	https://www.graphpad.com/scientific-software/prism/
Chimera	Pettersen et al.^53^	https://www.cgl.ucsf.edu/chimera/

### Resource availability

#### Lead contact

Further information and requests for resources and reagents should be directed to and will be fulfilled by the lead contact, Peter D. Kwong (pdkwong@nih.gov).

#### Materials availability

Plasmids generated in this study are available upon request.

### Experimental model and study participant details

#### Cell lines

The EXPI 293 and FreeStyle 293F cell lines were obtained from Thermo Fisher.

### Method details

#### High-throughput ELISA screening

2.5 x 10^4^ log-phase HEK 293T cells in 100 μL of RealFect Expression medium (ABI Scientific, VA) per well were inoculated in a 96-well cell culture microplate (Corning Scientific, NY) and allowed to grow for 24 h at 37°C, 5% CO_2_. Immediately before transfection, 40 μL of spent medium per well was removed. 250 ng of plasmid DNA encoding a HIV-1 trimer variant (GenScript synthesized) in 10 μL of Opti-MEM Reduced Serum medium (Thermo Fisher Scientific, CA) was mixed with 0.75 μL of TrueFect Max transfection reagent (United Biosystems, MD) in 10μL of Opti-MEM Reduced Serum medium at room temperature (RT) for 15 min, then mixed with growing cells per well in 96-well cell culture microplate. Transfected cells were incubated at 37°C and 5% CO_2_ for overnight (about 15 h), and then fed with 25 μL per well of CelBooster medium (Cell Growth Enhancer for adherent cells, ABI Scientific) with additional 3x Streptomycin-Penicillin and 10% FBS. Four days post transfection, the supernatant in the cell well was harvested, and analyzed in a 96-well plate formatted ELISA. Briefly, 96-well ELISA plates (Nunc Maxisorp, Thermo Fisher Scientific) were coated with 100 μL per well of Lectin at a concentration of 5 μg/mL (Galanthus Nivalis, SIGMA) in PBS overnight at 4°C, followed by blocking with a standard block solution (1% BSA and 0.05% Tween in PBS). 30 μL of expression supernatant and 70 μL of PBS per well were incubated in Lectin-coated plate at RT for 2 h. Captured trimer proteins were characterized by incubating with various primary antibodies at a concentration of 10 μg/mL at RT for 60 min, followed by detecting bound primary antibodies with anti-human IgG Fc HRP-conjugate (Jackson ImmunoResearch Labs, PA) at RT for 30 min. After final washing, the reaction signal was detected by addition of 100 μL per well of BioFX-TMB (SurModics, MN) at RT for 10 min. The reaction was stopped by addition of 100 μL per well of 0.5N H2SO4. The signal was measured at 450nm wavelength on a microplate reader (SpectraMax Plus, Molecular Devices, CA).

#### HIV Env expression and purification

HIV Env trimers were purified in a manner as previously described for SARS-CoV2-S2P probes.^54^ The Env trimers, genetically fused to a single-chain Fc domain, were transiently transfected into 293Freestyle cells and allowed to grow for 5 days at 37°C. The protein was purified from the supernatant using Protein A Sepharose Fast Flow resin (Cytivia), and the tag cleaved using HRV-3C protease. The collected trimer was applied to a Superdex S-200 gel filtration column equilibrated in PBS, pH 7.4. After gel filtration, the peak containing the HIV trimer was concentrated and supplemented with 10% glycerol, flash frozen in liquid nitrogen, and stored at −80°C until use.

#### Fusion peptide-conjugate immunogens

The FP8v1-rTTHC conjugate was produced by coupling the FP8v1 peptide sequence appended with a C-terminal cysteine (AVGIGAVF-C) to recombinant tetanus toxoid heavy chain fragment (rTTHC) using a sulfosuccinimidyl(4-iodoacetyl)aminobenzoate (sulfo-SIAB) heterobifunctional crosslinker (VRC Production Program).^31^ The conjugation ratio of peptide to carrier protein for the FP8v1-rTTHC conjugate was 5.4, as determined by amino acid analysis.^55^

#### Differential scanning calorimetry

DSC scans were performed on the HIV trimers using a Microcal VP-DSC instrument (GE Healthcare/MicroCal). Protein samples at 0.25 mg/ml were loaded and heated from 20°C to 95 °C at a rate of 1°C per minute. Melting temperatures (T_m_) were calculated using the included Origin software.

#### N-glycan profiling and occupancy by LC-MS/MS for gp41 and introduced sites

An LC-MS/MS^E^ method was used to determine the glycosylation site occupancy and glycan profiles of the individual glycosites.^24^ Briefly, the purified samples were buffer-exchanged to 50 mM ammonium bicarbonate (J.T. Baker, Phillipsburg, NJ) denatured with RapiGest (Waters, Milford, MA), reduced with DTT (ThermoFisher Life Technologies, Grand Island, NY). Four complementary proteolytic digests were applied using trypsin, chymotrypsin, LysC, and a mixture of trypsin with chymotrypsin (New England Biolab, Ipswich, MA). The portion of the resulting digests was deglycosylated with a mixture of PNGase F, Endo H (Promega, Madison, WI), and alpha 1-2,3 mannosidase (New England Biolab, Ipswich, MA). The digests were a subject for RPLC separation on an Acquity H-Class chromatography system with MS/MS^E^ analysis of glycan structures on an SYNAPT G2 QTof mass spectrometer, both from Waters (Milford, MA). The data processing was performed using BiopharmaLynx followed by manual inspection of the MS/MS spectra. Glycan occupancy was estimated based on the relative amounts of the non-modified and deamidated components resulting from the deglycosylation.

#### Glycopeptide identification and determination of occupancy of N-glycan sites in gp120

For N-glycan occupancy analyses, 10 μg of each intact gp120 or gp120 after N-glycans were removed by peptide-N-glycosidase F (PNGase F) (Prozyme, Hayward, CA) was loaded onto a 10% SDS-PAGE under denaturing and reducing conditions. The bands (∼130 kDa for intact, and ∼65 kDa for PNGase F-treated, fully deglycosylated) were excised, digested with trypsin or chymotrypsin (Promega), extracted from the gel matrix, and used for high-resolution mass spectrometry analysis.

LC-MS and MS/MS analysis of gp120 was carried out as previously described.^56^ Briefly, peptide/glycopeptide mixtures extracted from the SDS-PAGE were analytically separated on a self-prepared C18 reversed phase column via a nano-liquid chromatography (nano-LC) system. The eluted peptides were electrosprayed at 2 kV into a dual linear quadrupole ion trap Orbitrap Velos Pro mass spectrometer (Thermo Fisher, San Jose, CA). The mass spectrometer was set to switch between a full scan (400 < m/z < 2,000) followed by successive MS/MS (200 < m/z < 2,000) scans of the 10 most abundant precursor ions (parent ions) using the collision-induced dissociation (CID) method.

All LC-MS/MS data for deglycosylated gp120 digested with proteases were analyzed by the use of Optys Pinnacle software (version 1.0.103 Optys Tech Corporation). Peptide identification was achieved using the Single Protein Screening and Quantitation workflow with a peptide tolerance of 10 ppm in MS1 and an MS/MS tolerance of 0.7 Da. All peptide assignments were manually validated.

The presence of N-glycosylation in the intact gp120 was inferred by the increase of 1 Da in the peptide mass value of the deglycosylated gp120 because PNGase F treatment converted each glycosylated Asn into Asp. The % occupancy of each N-glycan site was calculation based on the areas under the peak for the peptide containing the unmodified Asn (the amount of unglycosylated) and the peptide containing the Asp (the amount of glycosylated).

#### Surface plasmon resonance

Surface Plasmon Resonance analysis was performed to determine the affinity of the purified HIV trimers to soluble CD4 (sCD4) using a Biacore T-200 instrument (GE Healthcare). 2G12 antibody was immobilized onto a CM5 chip to approximately 2,000 RU, after which the HIV trimers were loaded onto the immobilized antibody. sCD4 was injected at five concentrations (30-500nM) incrementally in a single cycle configuration at 50 μL/min every 60 s, followed by a dissociation phase of 30 min. All loading and binding phases were performed in HBP-EP+ buffer (10 mM HEPES pH 7.4, 150 mM NaCl, 3 mM EDTA, and 0.05% P-20) Binding curves of the injection series were corrected with corresponding blank channels and fit using the Biacore T-200 Evaluation software. Related to CD4 affinity, we note that current data indicates that alteration in CD4 affinity inversely relates to the stability of the pre-fusion closed conformation of the Env trimer. This effect has been previously reported with fewer stabilizations than reported here, decreasing affinity from 1 nM to 400 nM with increasing modifications.^22^

#### Antigenic analysis of Env trimers by MSD-ECLIA

Standard 96-well bare Multi-Array plates (catalog no. L15XA-3; MSD) were coated with all or a subset of a panel of HIV-neutralizing (VRC01, b12, VRC13, PGT121, PGT128, 2G12, PGT145, CAP256-VRC26.25, 35O22, 8ANC195, PGT151, and VRC34.01 and non-neutralizing or weakly neutralizing monoclonal (F105, 17b [± soluble CD4 {sCD4}], 48D [±sCD4], 447-52D [±sCD4], 3074 [±sCD4], 2557 [±sCD4]) and base binding antibodies (1E6, 989, 5H3, 3H2) noncognate (anti-influenza antibody Mota IgG) antibodies in duplicate (30 μL/well) at a concentration of 4 μg/mL diluted in 1× PBS by incubating overnight at 4°C. The following day, the plates were washed (wash buffer, 0.05% Tween 20 plus 1× PBS) and blocked with 150 μL of blocking buffer (5% [wt/vol] blocker A [catalog no. R93BA-4; MSD]) by incubating for 1 h on a vibrational shaker (Heidolph Titramax 100, catalog no. 544-11200-00) at 650 rpm. All incubations were performed at room temperature except for the coating step. During the incubation, trimers were titrated in serial 2× dilutions starting at a concentration of 5 μg/mL of the trimer in the assay diluent (1% [wt/vol] MSD blocker A plus 0.05% Tween 20). For sCD4 induction, the trimer was combined with sCD4 at a constant molar concentration of 1 μM before being added to the MSD plate. After the incubation with blocking buffer was complete, the plates were washed, and the diluted trimer was transferred (25 μL/well) to the MSD plates and incubated for 2 h on the vibrational shaker at 650 rpm. After the 2-h incubation with trimer, the plates were washed again and 2G12 antibody labeled with Sulfo-Tag (catalog no. R91AO-1; MSD) at a conjugation ratio of 1:15 (2G12: Sulfo-Tag), which was diluted in assay diluent at 4 μg/mL, added to the plates (25 μL/well), and incubated for 1 h on the vibrational shaker at 650 rpm. The plates were washed and read using read buffer (Read Buffer T, catalog no. R92TC-1; MSD) on the MSD Sector Imager 600 or equivalent instrument.

#### Cryo-EM single particle analysis of glycan-base ConC

Glycan-base ConC trimer at a concentration of 5 mg/mL was supplemented with 0.01% DDM and frozen onto Quantifoil R 2/2 grids using a FEI Vitrobot Mark IV plunger at 4°C and 95% relative humidity. Datasets were collected at NICE cryo-EM facility on an FEI Titan Krios electron microscope equipped with a Gatan K3 summit DED operated in the super-resolution mode (pixel size before binning: 0.415 Å). Cryosparc 3.3^52^ was used for CTF, 2D classifications, *ab initio* 3D reconstructions, homogeneous and non-uniform refinements. Initial reconstructions were performed with C1 symmetry, before moving to C3 symmetry for the final maps. The structure of the protein-base ConC (PDB 6CK9) was docked into the map, and refined by alternating rounds of manual building in WinCoot^50^ and automated refinement in Phenix.^49^ Figures were generated using Pymol and Chimera.^53^

#### Animal protocols and immunizations

All animal experiments were reviewed and approved by the Animal Care and Use Committee of the Vaccine Research Center (VRC), NIAID, NIH. Animals were housed and cared for in accordance with local, state, federal, and institute policies in an American Association for Accreditation of Laboratory Animal Care-accredited facility at the VRC.

For mouse studies, female C57BL/6 mice around 8 weeks old (Jackson Laboratory, Wilmington, MA) were immunized in two-week intervals for FP-primed regimens or in three-week intervals for trimer-only immunization regimens. For each immunization, 25 μg HIV-1 Env trimer or 25 μg FP8v1-rTTHC conjugate immunogens were formulated with 20% (v/v) Adjuplex adjuvant (research-grade Adjuplex, Advanced BioAdjuvants LLC of Omaha (ABA)) in a final injection volume of 100 μL. Cocktails of FP8v1-rTTHC and BG505 Env trimer were prepared by mixing 25 μg of each immunogen and cocktails of BG505 and ConC trimers were prepared by mixing 12.5 μg of each trimer prior to diluting in PBS with 20% (v/v) Adjuplex. Immunizations were administered intramuscularly as two separate injections of 50 μL each to the caudal thigh muscle of the two hind legs. Sera samples were collected two weeks after each immunization for serological analyses.

For guinea pig studies, two-month-old female Hartley guinea pigs with body weights of 300g (Charles River Laboratories, Wilmington, MA) were immunized every four weeks. For each immunization, 25 μg of HIV-1 Env trimer was diluted in PBS with 20% (v/v) Adjuplex (80 μL of research-grade Adjuplex, Advanced BioAdjuvants LLC of Omaha (ABA)) in a final volume of 400 μL. Immunizations were given intramuscularly as two separate injections of 200 μL into each quadriceps muscle. Sera samples were collected for serological analyses two weeks following each immunization.

#### Neutralization assays

Neutralization assays were performed using single round of infection HIV-1 Env-pseudoviruses and TZM-bl target cells, as previously described.^28^^,^^57^ The Δ611 mutant of BG505 is especially sensitive to FP-directed neutralization and was used to assess FP-directed responses. Neutralization curves were fit by nonlinear regression using a 5-parameter hill slope equation. The 50% and 80% inhibitory dilutions (ID50 and ID80) were reported as the reciprocal of the dilutions required to inhibit infection by 50% and 80%, respectively. Single-point assays were performed in duplicate at a dilution of 1:50, and data reported as percent neutralization.

#### Enzyme-linked immunosorbent assay (ELISA)

Anti-trimer ELISA were performed using lectin-captured HIV-1 trimers, as previously described.^28^ Ninety-six-well plates (Costar High Binding Half-Area; Corning, Kennebunk, ME) were coated overnight at 4°C with 50 μL/well snowdrop lectin from *Galanthus nivalis* (Sigma-Aldrich, St. Louis, MO) in PBS. Plates were washed five times with PBS-T (PBS plus 0.05% Tween) between each subsequent step. After being coated, plates were blocked with 100 μL/well of blocking buffer (5% skim milk in PBS) and incubated at room temperature for 60 min, followed by trimer capture with 2 μg/mL HIV-1 trimers in 10% FBS-PBS for 2 h at room temperature. Next, 50 μL/well serially diluted sera (5-fold; starting dilution of 1:100 or 1:1000) in 0.2% Tween-PBS buffer was added and incubated for 1 h at room temperature. Following incubation, goat anti-guinea pig IgG conjugated to horseradish peroxidase (Invitrogen, Waltham, MA) diluted 1:5,000 in 5% skim milk (BD Life Sciences, Sparks, MD) PBS buffer or goat anti-mouse IgG conjugated to horseradish peroxidase (Invitrogen, Waltham, MA) diluted 1:2,000 in 5% skim milk (BD Life Sciences, Sparks, MD) PBS buffer was added at 50 μL/well for 60 min at room temperature. Plates were washed five times with PBS-T and developed with 50 μL/well tetramethylbenzidine (TMB) substrate (SureBlue; KPL, Gaithersburg, MD) for 10 min at room temperature before the addition of 50 μL/well 1 N sulfuric acid (Fisher Chemical, Fair Lawn, NJ), without washing, to stop the reaction. Plates were read at 450 nm (SpectraMax using SoftMax Pro, version 5, software; Molecular Devices, Sunnyvale, CA), and optical densities (OD) were analyzed following subtraction of the nonspecific horseradish peroxidase background activity. The endpoint titer was defined as the reciprocal of the greatest dilution with an OD value above 0.1 (2 times average raw plate background).

#### Preparation of HIV-1 Env-Fab complexes for EMPEM

1 mg of total Fab from immunized guinea pigs was incubated overnight with 10–20 μg HIV-1 Env trimers at room temperature. Env-Fab complexes were purified by size exclusion chromatography using a Superose 6 Increase 10/300 column (Cytiva) in a buffer containing 150 mM NaCl and 5 mM HEPES, pH 7.4. The fractions containing the Env-Fab complexes were pooled and concentrated to ∼1 mg/mL.

#### Negative-stain EM

The HIV-1 Env or purified Env-Fab complexes were diluted to achieve a trimer concentration of approximately 0.02 mg/mL, 4.8 μL of the diluted protein solution was adsorbed to freshly glow-discharged carbon-coated grids for ∼15 s, then dried with wick-paper and rinsed 3 times with 4.8 μL of buffer containing 10 mM HEPES, pH 7.0, and 150 mM NaCl before stained with 0.75% uranyl formate for 30 s. Datasets were collected using a Thermo Scientific Talos F200C transmission electron microscope operated at 200 kV and equipped with a Ceta camera. The nominal magnification was 57,000×, corresponding to a pixel size of 2.53 Å, and the defocus was set at −1.2 μm. For EMPEM datasets, micrographs were collected to ensure more than 100,000 single particles could be picked. Particle-picking, 2D and 3D classification were carried out using CryoSparc 3.3.^52^

### Quantification and statistical analysis

For Figure 1B, averaging the area under the curve for sera response, bar height indicates the mean, and error bars represent the standard deviation, for 3 animals in each case (represented by data points). Similarly, for ELISA and neutralization data displayed in Figures 5E and 5F, the mean and standard deviation are displayed including 10 animals per group, and the p-value is calculated using the Mann-Whitney nonparametric test. Finally in the case of the guinea pig ELISA data displayed in Figures 6C and 7C, the Mann-Whitney nonparametric test is used for p-value determination, and geometric mean and 95% confidence interval is shown for 10 animals per group.

## Data Availability

• Cryo-EM reconstruction maps and refined atomic structures have been deposited to the Electron Microscopy Data Bank (EMDB: EMD-28910) and Protein Data Bank (PDB: 8F7T), respectively, and are publicly available as of the date of publication. Accession numbers are listed in the key resources table. • This paper does not report original code. • Any additional information required to reanalyze the data reported in this paper is available from the lead contact upon request.
