# Supplementary material for: Soluble prefusion-closed HIV-envelope trimers with glycan-covered bases
Source: iScience. 2023 Jul 15;26(8):107403. doi: 10.1016/j.isci.2023.107403 (PMC10404741; doi:10.1016/j.isci.2023.107403)
Supplement: Document S1. Figures S1–S6 and Tables S1–S4 [file mmc1.pdf]

iScience, Volume *26*

Supplemental information

Soluble prefusion-closed HIV-envelope trimers with glycan-covered bases

Adam S. Olia, Cheng Cheng, Tongqing Zhou, Andrea Biju, Darcy R. Harris, Anita Changela, Hongying Duan, Vera B. Ivleva, Wing-Pui Kong, Li Ou, Reda Rawi, Yaroslav Tsybovsky, David J. Van Wazer, Angela R. Corrigan, Christopher A. Gonelli, Myungjin Lee, Krisha McKee, Sandeep Narpala, Sijy O'Dell, Danealle K. Parchment, Erik-Stephane D. Stancofski, Tyler Stephens, Ivy Tan, I-Ting Teng, Shuishu Wang, Qing Wei, Yongping Yang, Zhengrong Yang, Baoshan Zhang, VRC Production Program, Jan Novak, Matthew B. Renfrow, Nicole A. Doria-Rose, Richard A. Koup, Adrian B. McDermott, Jason G. Gall, Q. Paula Lei, John R. Mascola, and Peter D. Kwong

Table S1. Antigenic screening of base-covered trimer designs, related to Figure 1. (A) Original high-throughput ELISA data. Color scale ranges from white (lowest value) to red (highest value). (B) ELISA raw data values normalized by the reference strain BG505-RnS(7MutV1)-2G-3mut-DS_SOSIP. We consider a trimer to be in the prefusion-closed conformation if either VRC26.25, PGT145, or PGDM1400 binding is greater 0.75.

A

Trimer Construct	VRC26.25	PGT145	PGDM1400	PGT151	35O22	VRC34	PGT122	N6	3BNC117	F105	447-52D	17b	17b+CD4	1E6	5H3	3H2	989	RM20A3
BG505-RnS(7MutV1)-2G-3mut-DS_SOSIP-scFc	0.7097	0.6834	0.9852	1.2956	1.0098	0.783	1.0493	0.811	1.0396	0.1344	0.5004	0.0844	0.0858	3.013	2.8426	3.0678	0.1953	2.6452
BG505-RnS(7MutV1)-2G-3mut-DS_SOSIP-502-660-NxT-scFc	0.6698	0.5903	1.1126	0.8404	0.9137	0.5669	1.0121	0.8165	1.0522	0.1661	0.5486	0.0743	0.0719	1.9949	1.3055	0.2111	0.1094	2.0276
BG505-DSSOSIP (non-scFc)	0.2128	0.1514	0.3322	1.1526	0.296	0.4572	0.3242	0.5538	0.6036	0.1288	0.4464	0.0513	0.052	1.6706	1.6523	1.6086	0.4052	1.4786
BG505.RnS7Mut-N28-502-660	0.5948	0.393	1.0888	0.5863	0.8204	0.4681	0.809	0.7076	0.8509	0.1088	0.4727	0.0728	0.0703	1.3954	0.6765	0.143	0.1629	1.156
BG505.RnS7Mut-502-660-665	0.5272	0.508	1.0459	0.9529	0.7867	0.4609	0.8615	0.6712	0.8376	0.1029	0.4661	0.0778	0.0688	0.6948	0.3487	0.123	0.1146	1.0426
BG505.RnS7Mut-N28-502-660-665	0.5547	0.531	0.9816	0.5018	0.7854	0.4367	0.8638	0.6656	0.8413	0.1062	0.4534	0.0697	0.0668	0.5019	0.2292	0.1062	0.1	0.6078
BG505.RnS7Mut-JCBv1-660	0.6878	0.521	0.9719	0.784	0.9439	0.7315	0.8651	0.7033	0.9533	0.1019	0.4163	0.0716	0.0699	1.9946	2.6564	0.9006	0.1063	2.686
BG505.RnS7Mut-JCBv2-660	0.5914	0.3345	1.03	0.7853	0.7394	0.605	0.8946	0.7067	0.9769	0.1203	0.4631	0.0828	0.0681	0.3602	0.7243	0.1258	0.1159	1.4536
BG505.RnS7Mut-JCBv3-660	0.5882	0.2766	0.9981	0.5759	0.7495	0.6638	0.8472	0.6668	0.9391	0.1239	0.4478	0.0695	0.0676	0.3059	0.4952	0.1229	0.1204	1.0324
BG505.RnS7Mut-N28-JCBv1-660	0.7449	0.6327	0.9999	0.6327	0.8412	0.726	0.8993	0.7154	0.9165	0.1108	0.4409	0.0748	0.0707	2.1004	2.6831	0.8311	0.1153	2.7296
BG505.RnS7Mut-N28-JCBv2-660	0.6286	0.2708	1.0573	0.4196	0.7241	0.6901	0.8906	0.7758	0.9536	0.1305	0.4757	0.1091	0.0829	0.2692	0.3307	0.1107	0.1175	0.6388
BG505.RnS7Mut-N28-JCBv3-660	0.6523	0.4267	1.0882	0.428	0.7281	0.6479	0.9061	0.7712	1.0124	0.1962	0.505	0.0732	0.0876	0.2594	0.3061	0.1105	0.1459	0.6193
BG505.RnS7Mut-JCBv1-660-665	0.6711	0.4485	1.0172	0.6906	0.9306	0.747	0.7197	0.7396	0.936	0.1105	0.469	0.0699	0.0882	0.9778	1.8077	0.2431	0.1178	2.0142
BG505.RnS7Mut-JCBv2-660-665	0.1354	0.1982	0.9484	0.2104	0.2585	0.2135	0.2454	0.6594	0.7344	0.0866	0.4569	0.0679	0.0767	0.0953	0.3004	0.768	0.8046	0.396
BG505.RnS7Mut-JCBv3-660-665	0.5267	0.2007	1.0799	0.3515	0.6331	0.6907	0.8136	0.8107	0.9648	0.1276	0.477	0.07	0.066	0.1835	0.1102	0.0997	0.1021	0.2524
BG505.RnS7Mut-N28-JCBv1-660-665	0.7183	1.0318	0.9555	0.9673	0.8729	0.7556	1.035	0.7257	0.8793	0.1089	0.4342	0.0704	0.0668	1.2531	2.4027	3.2213	1.0854	2.7133
BG505.RnS7Mut-N28-JCBv2-660-665	0.5437	0.2726	1.0263	0.3824	0.6279	0.7288	1.005	0.8161	0.9521	0.1407	0.4751	0.0715	0.0681	0.1913	0.1187	0.1024	0.1011	0.2581
BG505.RnS7Mut-N28-JCBv3-660-665	0.564	0.2189	1.0685	0.2939	0.6323	0.6824	0.8744	0.7951	0.9812	0.1567	0.4775	0.0701	0.0659	0.158	0.0949	0.1024	0.0977	0.2012
BG505 DSSOSIP-N332-RnS7Mut-502-660	0.5186	0.3331	0.9908	0.8651	0.8035	0.4597	0.8017	0.6875	0.8468	0.1025	0.4613	0.1153	0.0668	1.6257	0.9672	0.1703	0.1053	1.6221
scFc-3C-6931-502-660	0.7697	0.6993	0.502	0.465	0.8538	0.738	1.3564	0.8689	1.0564	0.311	0.3629	0.0637	0.2196	0.1526	0.0731	0.1129	0.1114	0.0766
scFc-3C-6931-DS-502-660	0.6966	0.5988	0.5125	0.3859	0.7938	0.6726	1.2645	0.7923	0.9698	0.3036	0.3904	0.0641	0.0637	0.1429	0.0842	0.1026	0.1043	0.0755
scFc-3C-6931-28-502-660	0.4774	1.2607	0.3632	0.8113	0.68	0.8345	1.1005	0.9527	0.7035	0.3625	0.2506	0.0723	0.3282	0.2895	0.0943	0.0934	0.1136	0.0848
scFc-3C-6931-502-660-665	0.8116	1.0057	0.8343	0.5222	0.907	0.755	1.4531	0.9658	1.1235	0.4813	0.496	0.084	0.3054	0.1463	0.0814	0.1085	0.1187	0.088
scFc-3C-6931-28-502-660-665	0.9092	1.7704	0.8241	0.6161	0.9075	0.8133	1.5308	0.9949	1.1625	0.4106	0.44	0.082	0.3877	0.1418	0.0952	0.1124	0.1381	0.0923
scFc-3C-6931-JCB04V1-660	0.9524	1.2986	0.7995	0.6959	1.0953	1.0574	1.4368	0.9716	1.1739	0.3626	0.4426	0.0764	0.2371	0.1508	0.0857	0.1262	0.1088	0.0831
scFc-3C-6931-JCB04V2-660	0.8474	0.9306	0.7377	0.9122	0.9467	0.9947	1.4316	0.9781	1.1563	0.4077	0.4549	0.0877	0.3433	0.1525	0.0879	0.1569	0.1143	0.0806
scFc-3C-6931-JCB04V3-660	0.845	1.0386	0.8166	0.8023	0.9057	0.984	1.4554	1.0396	1.1858	0.4802	0.4661	0.1221	0.3614	0.1743	0.0811	0.1092	0.1202	0.0806
scFc-3C-6931-28-JCB04V1-660	1.0692	1.5232	0.8676	0.7782	1.0882	1.0422	1.4268	0.9507	1.1363	0.3353	0.3784	0.0787	0.197	0.1752	0.0883	0.1199	0.1158	0.086
scFc-3C-6931-28-JCB04V2-660	0.806	0.9723	0.7066	0.7829	0.8735	1.0008	1.4242	0.9782	1.1305	0.3967	0.4054	0.0771	0.3483	0.1585	0.0881	0.1151	0.1167	0.0842
scFc-3C-6931-28-JCB04V3-660	0.8551	0.8257	0.7578	0.5603	0.859	0.9511	1.443	0.9774	1.1679	0.412	0.393	0.0781	0.3959	0.1591	0.0749	0.1116	0.1074	0.0739
scFc-3C-6931-JCB04V1-660-665	0.908	1.3799	0.8096	1.3173	0.9663	0.9674	1.4047	0.9127	1.1648	0.3975	0.4353	0.1078	0.2654	0.1398	0.0788	0.1096	0.1253	0.0794
scFc-3C-6931-JCB04V2-660-665	0.6743	0.6982	0.7076	0.7349	0.7827	0.8605	1.4582	0.9489	1.1616	0.4747	0.4623	0.0753	0.4504	0.1548	0.0752	0.1129	0.124	0.0788
scFc-3C-6931-JCB04V3-660-665	0.6345	0.6578	0.7871	0.5087	0.7383	0.846	1.3991	1.0121	1.2297	0.473	0.4644	0.0743	0.4738	0.1493	0.0752	0.1128	0.1339	0.0923
scFc-3C-6931-28-JCB04V1-660-665	0.9788	1.7291	0.7754	1.5497	0.9683	0.9497	1.4591	0.919	1.1807	0.32	0.3591	0.0737	0.2384	0.1531	0.0865	0.1253	0.1321	0.0895
scFc-3C-6931-28-JCB04V2-660-665	0.5581	0.4141	0.4386	0.3994	0.6937	0.7607	1.343	0.9256	1.1841	0.3082	0.3287	0.0938	0.4293	0.1556	0.0723	0.1087	0.1145	0.0798
scFc-3C-6931-28-JCB04V3-660-665	0.5106	0.334	0.5233	0.312	0.6152	0.8282	1.3411	0.9903	1.1907	0.4025	0.3553	0.0871	0.4855	0.1415	0.0732	0.1077	0.1086	0.0871
scFc-3C-6931-DS-28-502-660	0.9518	1.2915	0.8805	0.6124	0.9488	0.8195	1.452	0.9204	1.0959	0.375	0.4862	0.0758	0.0724	0.1606	0.1332	0.1264	0.1313	0.0859
scFc-3C-6931-DS-502-660-665	0.788	0.7999	0.8094	0.4164	0.9312	0.6847	1.3247	0.9238	1.0384	0.3906	0.5016	0.0754	0.0684	0.1449	0.0812	0.1136	0.1118	0.0747
scFc-3C-6931-DS-28-502-660-665	0.8741	0.9377	0.6806	0.4116	0.9158	0.7177	1.3601	0.8761	1.0321	0.337	0.4179	0.0731	0.0699	0.1406	0.0768	0.1033	0.1014	0.0753
scFc-3C-6931-DS-JCB04V1-660	0.8053	0.6727	0.5861	1.7975	0.9671	0.8951	1.243	0.7811	0.9504	0.2849	0.3701	0.0682	0.0651	0.1881	0.0811	0.145	0.0968	0.0725
scFc-3C-6931-DS-JCB04V2-660	0.7453	0.5572	0.5258	0.4395	0.849	0.8958	1.2681	0.8406	1.0071	0.3455	0.4091	0.0708	0.0685	0.1665	0.1152	0.1651	0.1896	0.1114
scFc-3C-6931-DS-JCB04V3-660	0.6182	0.5088	0.4959	0.3458	0.8476	0.7919	1.1699	0.8103	0.9775	0.3042	0.3496	0.0672	0.0627	0.1453	0.0658	0.1053	0.0889	0.0658
scFc-3C-6931-DS-28_JCB04V1-660	0.9052	1.2647	0.8457	0.6332	0.9922	0.9677	1.4341	0.8805	1.1198	0.3622	0.4287	0.0752	0.0691	0.1464	0.0834	0.1238	0.1154	0.08
scFc-3C-6931-DS-28_JCB04V3-660	0.9794	1.2584	0.8607	0.5043	0.9002	0.8896	1.4298	0.9181	1.1328	0.4322	0.4611	0.0742	0.0699	0.1649	0.076	0.1185	0.1162	0.0805
scFc-3C-6931-DS-JCB04V1-660-665	0.8376	1.0525	0.7265	0.7834	0.9411	0.9287	0.9936	0.867	0.9938	0.4003	0.4349	0.07	0.0592	0.146	0.0685	0.1136	0.1155	0.0816
scFc-3C-6931-DS-JCB04V2-660-665	0.7509	0.9481	0.8203	0.5039	0.8217	0.8436	1.3883	0.9446	1.1421	0.4741	0.5293	0.0719	0.0689	0.1522	0.0843	0.1154	0.1202	0.0788
scFc-3C-6931-DS-JCB04V3-660-665	0.8378	0.8729	0.8358	0.4397	0.7868	0.8355	1.4086	0.9668	1.146	0.4574	0.5231	0.0713	0.0719	0.139	0.079	0.1084	0.1169	0.0838
scFc-3C-6931-DS-28_JCB04V1-660-665	0.9944	1.881	0.8694	0.9803	0.9634	1.0202	1.5422	0.9481	1.1054	0.4205	0.466	0.0775	0.0711	0.1502	0.1012	0.1352	0.1399	0.1152
scFc-3C-6931-DS-28_JCB04V2-660-665	0.7542	1.5244	0.9254	0.6607	0.9833	1.0015	1.4798	1.6166	1.4176	0.6003	0.6984	0.1336	0.1028	0.1783	0.1099	0.1403	0.1591	0.0884
scFc-3C-6931-DS-28_JCB04V3-660-665	0.7984	1.2384	0.9488	0.5197	0.9541	1.0469	1.4843	1.5614	1.4305	0.6306	0.7274	0.1045	0.1083	0.1567	0.1169	0.1174	0.121	0.0863
Control	0.0684	0.0536	0.0561	0.1847	0.0761	0.0556	0.1206	0.0814	0.0733	0.0679	0.1596	0.0511	0.0525	0.1352	0.0555	0.1087	0.0804	0.0628
scFc-BG505-RnS(5Mut)-DS-SOSIP-2G-3Mut-502-660-NxT (T010-502-660)	0.5026	0.1526	1.0006	0.3362	0.9349	0.6038	0.4496	1.2567	1.2172	0.1625	0.6591	0.0996	0.0974	0.6592	0.3446	0.096	0.073	0.4159
T010 (BG505-RnS(5Mut)-DS-SOSIP-2G-3Mut)	0.5135	0.7531	1.0917	1.5268	0.84	0.753	1.1286	1.2283	1.2208	0.195	0.6776	0.0916	0.0943	2.9584	2.8771	3.0547	0.3312	2.669

B

Trimer Construct	VRC26.25	PGT145	PGDM1400	PGT151	VRC34	35O22	PGT122	N6	3BNC117	F105	447-52D	17b	17b+CD4	1E6	5H3	3H2	989	RM20A3
BG505-RnS(7MutV1)-2G-3mut-DS_SOSIP-scFc	1	1	1	1	1	1	1	1	1	1	1	1	1	1	1	1	1	1
BG505-RnS(7MutV1)-2G-3mut-DS_SOSIP-502-660-NxT-scFc	0.943779	0.863769	1.129314	0.648657	0.72401	0.904833	0.964548	1.006782	1.01212	1.235863	1.096323	0.880332	0.837995	0.662098	0.459263	0.068812	0.560164	0.76652
BG505-DSSOSIP (non-scFc)	0.299845	0.221539	0.33719	0.889626	0.583908	0.293127	0.308968	0.682861	0.580608	0.958333	0.892086	0.60782	0.606061	0.554464	0.581264	0.52435	2.074757	0.558975
BG505.RnS7Mut-N28-502-660	0.838101	0.575066	1.105156	0.452532	0.597829	0.812438	0.77099	0.872503	0.818488	0.809524	0.944644	0.862559	0.819347	0.463126	0.237986	0.046613	0.834101	0.437018
BG505.RnS7Mut-502-660-665	0.742849	0.743342	1.061612	0.735489	0.588633	0.779065	0.821024	0.82762	0.805694	0.765625	0.931455	0.921801	0.801865	0.230601	0.122669	0.040094	0.58679	0.394148
BG505.RnS7Mut-N28-502-660-665	0.781598	0.776997	0.996346	0.387311	0.557727	0.777778	0.823215	0.820715	0.809254	0.790179	0.906075	0.825829	0.778555	0.166578	0.08063	0.034618	0.512033	0.229775
BG505.RnS7Mut-JCBv1-660	0.969142	0.762365	0.9865	0.605125	0.934227	0.93474	0.824454	0.867201	0.916987	0.758185	0.831934	0.848341	0.814685	0.661998	0.934497	0.293565	0.544291	1.015424
BG505.RnS7Mut-JCBv2-660	0.83331	0.489464	1.045473	0.606128	0.772669	0.732224	0.852568	0.871393	0.939688	0.895089	0.92546	0.981043	0.793706	0.119549	0.254802	0.041007	0.593446	0.549524
BG505.RnS7Mut-JCBv3-660	0.828801	0.404741	1.013094	0.444504	0.847765	0.742226	0.807395	0.822195	0.903328	0.921875	0.894884	0.82346	0.787879	0.101527	0.174207	0.040061	0.616487	0.390292
BG505.RnS7Mut-N28-JCBv1-660	1.049598	0.925812	1.014921	0.488345	0.927203	0.833036	0.857048	0.882121	0.881589	0.824405	0.881095	0.886256	0.824009	0.697113	0.943889	0.270911	0.590374	1.031907
BG505.RnS7Mut-N28-JCBv2-660	0.885726	0.396254	1.073183	0.323865	0.881354	0.717073	0.848756	0.956597	0.917276	0.970982	0.950639	1.292654	0.9662	0.089346	0.116337	0.036084	0.601639	0.241494
BG505.RnS7Mut-N28-JCBv3-660	0.919121	0.624378	1.104547	0.330349	0.827458	0.721034	0.863528	0.950925	0.973836	1.459821	1.009193	0.867299	1.020979	0.086094	0.107683	0.036019	0.747056	0.234122
BG505.RnS7Mut-JCBv1-660-665	0.945611	0.656277	1.032481	0.533035	0.954023	0.921569	0.685886	0.911961	0.900346	0.822173	0.93725	0.828199	1.027972	0.324527	0.635932	0.079242	0.603175	0.761455
BG505.RnS7Mut-JCBv2-660-665	0.190785	0.29002	0.962647	0.162396	0.272669	0.255991	0.23387	0.81307	0.706426	0.644345	0.91307	0.804502	0.893939	0.03163	0.105678	0.250342	4.119816	0.149705
BG505.RnS7Mut-JCBv3-660-665	0.742145	0.293679	1.096123	0.271303	0.88212	0.626956	0.775374	0.99963	0.928049	0.949405	0.953237	0.829384	0.769231	0.060903	0.038767	0.032499	0.522785	0.095418
BG505.RnS7Mut-N28-JCBv1-660-665	1.012118	1.509804	0.969854	0.746604	0.965006	0.864429	0.986372	0.894821	0.845806	0.810268	0.867706	0.834123	0.778555	0.415898	0.845247	1.050036	5.557604	1.025745
BG505.RnS7Mut-N28-JCBv2-660-665	0.766098	0.398888	1.041717	0.295153	0.930779	0.621806	0.957781	1.006289	0.915833	1.046875	0.94944	0.847156	0.793706	0.063492	0.041758	0.033379	0.517665	0.097573
BG505.RnS7Mut-N28-JCBv3-660-665	0.794702	0.32031	1.084551	0.226845	0.87152	0.626164	0.833317	0.980395	0.943825	1.165923	0.954237	0.830569	0.768065	0.052439	0.033385	0.033379	0.500256	0.076062
BG505 DSSOSIP-N332-RnS7Mut-502-660	0.730731	0.487416	1.005684	0.667722	0.587101	0.795702	0.764033	0.847719	0.814544	0.762649	0.921863	1.366114	0.778555	0.539562	0.340252	0.055512	0.539171	0.613224
scFc-3C-6931-502-660	1.084543	1.023266	0.509541	0.358907	0.942529	0.845514	1.292671	1.071393	1.01616	2.313988	0.72522	0.754739	2.559441	0.050647	0.025716	0.036802	0.570405	0.028958
scFc-3C-6931-DS-502-660	0.981541	0.876207	0.520199	0.297854	0.859004	0.786096	1.205089	0.976942	0.932859	2.258929	0.780176	0.759479	0.742424	0.047428	0.029621	0.033444	0.53405	0.028542
scFc-3C-6931-28-502-660	0.672679	1.844747	0.368656	0.626196	1.065773	0.673401	1.048794	1.174723	0.676703	2.697173	0.500799	0.856635	3.825175	0.096084	0.033174	0.030445	0.581669	0.032058
scFc-3C-6931-502-660-665	1.143582	1.471613	0.846833	0.403056	0.96424	0.898198	1.384828	1.190875	1.080704	3.581101	0.991207	0.995261	3.559441	0.048556	0.028636	0.035367	0.607783	0.033268
scFc-3C-6931-28-502-660-665	1.281105	2.590577	0.83648	0.475533	1.038697	0.898693	1.458877	1.226757	1.118219	3.05506	0.879297	0.971564	4.518648	0.047063	0.03349	0.036639	0.707117	0.034893
scFc-3C-6931-JCB04V1-660	1.341975	1.900205	0.81151	0.537126	1.350447	1.08467	1.369294	1.198027	1.129184	2.697917	0.884492	0.905213	2.763403	0.05005	0.030148	0.041137	0.557092	0.031415
scFc-3C-6931-JCB04V2-660	1.194026	1.361721	0.748782	0.704075	1.27037	0.937512	1.364338	1.206042	1.112255	3.033482	0.909073	1.0391	4.001166	0.050614	0.030922	0.051144	0.585253	0.03047
scFc-3C-6931-JCB04V3-660	1.190644	1.519754	0.828867	0.61925	1.256705	0.89691	1.38702	1.281874	1.140631	3.572917	0.931455	1.446682	4.212121	0.057849	0.02853	0.035596	0.615463	0.03047
scFc-3C-6931-28-JCB04V1-660	1.506552	2.228856	0.880633	0.600648	1.331034	1.077639	1.359764	1.172256	1.093017	2.494792	0.756195	0.932464	2.296037	0.058148	0.031063	0.039083	0.592934	0.032512
scFc-3C-6931-28-JCB04V2-660	1.135691	1.422739	0.717215	0.604276	1.278161	0.865023	1.357286	1.206165	1.087437	2.951637	0.810152	0.913507	4.059441	0.052605	0.030993	0.037519	0.597542	0.031831
scFc-3C-6931-28-JCB04V3-660	1.204875	1.208224	0.769184	0.432464	1.214687	0.850663	1.375203	1.205179	1.123413	3.065476	0.785372	0.925355	4.614219	0.052805	0.026349	0.036378	0.549923	0.027937
scFc-3C-6931-JCB04V1-660-665	1.279414	2.019169	0.821762	1.016749	1.235504	0.956922	1.338702	1.125401	1.120431	2.957589	0.869904	1.277251	3.09324	0.046399	0.027721	0.035726	0.641577	0.030017
scFc-3C-6931-JCB04V2-660-665	0.95012	1.021656	0.71823	0.567228	1.098978	0.775104	1.389688	1.170037	1.117353	3.531994	0.923861	0.89218	5.249417	0.051377	0.026455	0.036802	0.634921	0.02979
scFc-3C-6931-JCB04V3-660-665	0.89404	0.96254	0.798924	0.392637	1.08046	0.731135	1.333365	1.247965	1.182859	3.519345	0.928058	0.880332	5.522145	0.049552	0.026455	0.036769	0.685612	0.034893
scFc-3C-6931-28-JCB04V1-660-665	1.379174	2.530143	0.787048	1.196125	1.212899	0.958903	1.390546	1.133169	1.135725	2.380952	0.717626	0.873223	2.778555	0.050813	0.03043	0.040844	0.676395	0.033835
scFc-3C-6931-28-JCB04V2-660-665	0.786389	0.605941	0.445189	0.308274	0.97152	0.686968	1.279901	1.141307	1.138996	2.293155	0.656875	1.111374	5.003497	0.051643	0.025434	0.035433	0.586278	0.030168
scFc-3C-6931-28-JCB04V3-660-665	0.719459	0.488733	0.531161	0.240815	1.057727	0.60923	1.27809	1.221085	1.145344	2.994792	0.710032	1.031991	5.658508	0.046963	0.025751	0.035107	0.556068	0.032928
scFc-3C-6931-DS-28-502-660	1.34113	1.889816	0.893727	0.472677	1.046616	0.939592	1.38378	1.134895	1.054155	2.790179	0.971623	0.898104	0.843823	0.053302	0.046859	0.041202	0.672299	0.032474
scFc-3C-6931-DS-502-660-665	1.110328	1.170471	0.821559	0.321395	0.874457	0.922163	1.262461	1.139088	0.998846	2.90625	1.002398	0.893365	0.797203	0.048092	0.028565	0.03703	0.572453	0.02824
scFc-3C-6931-DS-28-502-660-665	1.231647	1.37211	0.690824	0.317691	0.916603	0.906912	1.296197	1.080271	0.992786	2.50744	0.835132	0.866114	0.814685	0.046664	0.027018	0.033672	0.519201	0.028467
scFc-3C-6931-DS-JCB04V1-660	1.134705	0.984343	0.594905	1.387388	1.143167	0.957714	1.184599	0.963132	0.914198	2.119792	0.739608	0.808057	0.758741	0.062429	0.02853	0.047265	0.495648	0.027408
scFc-3C-6931-DS-JCB04V2-660	1.050162	0.815335	0.533699	0.339225	1.144061	0.840761	1.20852	1.036498	0.968738	2.570685	0.817546	0.838863	0.798368	0.055261	0.040526	0.053817	0.970814	0.042114
scFc-3C-6931-DS-JCB04V3-660	0.871072	0.744513	0.50335	0.266903	1.011367	0.839374	1.114934	0.999137	0.940265	2.263393	0.698641	0.796209	0.730769	0.048224	0.023148	0.034324	0.455197	0.024875
scFc-3C-6931-DS-28_JCB04V1-660	1.275469	1.8506	0.858404	0.488731	1.235888	0.982571	1.366721	1.085697	1.077145	2.69494	0.856715	0.890995	0.805361	0.048589	0.029339	0.040355	0.590886	0.030243
scFc-3C-6931-DS-28_JCB04V3-660	1.38002	1.841381	0.87363	0.389241	1.136143	0.891464	1.362623	1.132059	1.08965	3.215774	0.921463	0.879147	0.814685	0.05473	0.026736	0.038627	0.594982	0.030432
scFc-3C-6931-DS-JCB04V1-660-665	1.180217	1.540094	0.737414	0.604662	1.186079	0.931967	0.946917	1.069051	0.955945	2.978423	0.869105	0.829384	0.689977	0.048457	0.024098	0.03703	0.591398	0.030848
scFc-3C-6931-DS-JCB04V2-660-665	1.058053	1.387328	0.832623	0.388932	1.077395	0.813725	1.323073	1.164735	1.098596	3.52753	1.057754	0.851896	0.80303	0.050514	0.029656	0.037617	0.615463	0.02979
scFc-3C-6931-DS-JCB04V3-660-665	1.180499	1.27729	0.848356	0.339379	1.06705	0.779164	1.342419	1.192109	1.102347	3.403274	1.045364	0.844787	0.837995	0.046133	0.027791	0.035335	0.598566	0.03168
scFc-3C-6931-DS-28_JCB04V1-660-665	1.401155	2.752414	0.88246	0.756638	1.302937	0.95405	1.469742	1.169051	1.063294	3.12872	0.931255	0.918246	0.828671	0.049851	0.035601	0.044071	0.716334	0.043551
scFc-3C-6931-DS-28_JCB04V2-660-665	1.062703	2.230612	0.939302	0.509957	1.279055	0.973757	1.410274	1.993342	1.363601	4.466518	1.395683	1.582938	1.198135	0.059177	0.038662	0.045733	0.814644	0.033419
scFc-3C-6931-DS-28_JCB04V3-660-665	1.124982	1.812116	0.963053	0.401127	1.337037	0.944841	1.414562	1.925277	1.37601	4.691964	1.453637	1.238152	1.262238	0.052008	0.041124	0.038268	0.61956	0.032625
Control	0.096379	0.078431	0.056943	0.142559	0.071009	0.075361	0.114934	0.10037	0.070508	0.505208	0.318945	0.60545	0.611888	0.044872	0.019524	0.035433	0.411674	0.023741
c-BG505-RnS(5Mut)-DS-SOSIP-2G-3Mut-502-660-NxT (T010-502-6	0.708187	0.223295	1.015631	0.259494	0.771137	0.925827	0.428476	1.549568	1.170835	1.209077	1.317146	1.180095	1.135198	0.218785	0.121227	0.031293	0.373784	0.157228
T010 (BG505-RnS(5Mut)-DS-SOSIP-2G-3Mut)	0.723545	1.10199	1.1081	1.17845	0.961686	0.831848	1.075574	1.51455	1.174298	1.450893	1.354117	1.085308	1.099068	0.981879	1.012137	0.99573	1.695853	1.008997

Table S2. Cryo-EM data collection, processing, and model refinement statistics for the glycan-base ConC structure, related to Figure 4.

	Glycan-base ConC
**EMDB ID**	EMD-28910
**PDB ID**	8F7T
Data Collection	
Microscope	FEI Titan Krios
Voltage (kV)	300
Electron dose (e^-/Å^2)	43.54
Detector	Gatan K3 BioQuantum
Pixel Size (Å)	0.415 / 0.830
Defocus Range (µm)	-0.7 / -2.0
Magnification	105,000
Reconstruction	
Software	cryoSPARC v3.3.1
Particles	12,967
Symmetry	C3
Box size (pix)	256
Resolution (Å) ($FSC_{0.143}$)	4.10
Refinement	
Software	Phenix 1.20
Protein residues	1641
Chimera CC	0.77
EMRinger Score	1.86
R.m.s. deviations	
Bond lengths (Å)	0.005
Bond angles (°)	0.746
Validation	
Molprobity score	2.26
Clash score	14.5
Favored rotamers (%)	99.1
Ramachandran	
Favored regions (%)	89.1
Allowed regions (%)	10.5
Disallowed regions (%)	0.4

Table S3. Week 6 neutralization titers (ID_{50}) for BG505 and ConC trimer-immunized groups in guinea pigs, related to Figures 6, 7.

A Week 6 serum neutralization titers (ID_{50}) against wildtype BG505, BG505.N611Q, MW965, HIV-2 7312A, and non-HIV strains, for protein-base and glycan-base BG505 trimer immunized groups in guinea pigs

Group	Clade / Virus / Animal ID	Non-HIV SIVmac251	Non-HIV SVA-MLV	Non-HIV 7312A_V434M	Non-HIV 7312A_V434M +sCD4	A BG505.W6M.C2. N611Q	A BG505.W6M.C2	C MW965.26
2108: Protein-base BG505	2108-1	<20	<20	<20	<20	834	69	627
	2108-2	<20	<20	<20	<20	721	313	590
	2108-3	<20	<20	<20	<20	260	<20	558
	2108-4	<20	<20	<20	<20	1,154	130	1,306
	2108-5	<20	<20	<20	<20	460	122	1,096
	2108-6	<20	<20	<20	<20	365	98	1,508
	2108-7	27	49	33	63	244	32	146
	2108-8	<20	<20	<20	<20	753	127	2,039
	2108-9	<20	<20	<20	<20	272	<20	245
	2108-10	<20	<20	<20	<20	2,808	2,600	246
2109: Glycan-base BG505	2109-1	<20	<20	<20	<20	<20	<20	38
	2109-2	<20	<20	<20	<20	74	20	1,039
	2109-3	<20	<20	<20	<20	<20	<20	54
	2109-4	<20	<20	<20	<20	139	72	2,739
	2109-5	<20	<20	<20	<20	<20	<20	441
	2109-6	<20	<20	<20	<20	<20	<20	508
	2109-7	<20	<20	<20	<20	<20	<20	1,047
	2109-8	<20	<20	<20	<20	<20	<20	413
	2109-9	<20	<20	<20	<20	<20	<20	654
	2109-10	<20	<20	<20	<20	<20	<20	212

10,000-100,000 | 1000-10,000 | 100-1000 | 50-100

B Week 6 serum neutralization titers (ID_{50}) against ConC, ConC.N611Q, MW965, and non-HIV strains, for protein-base and glycan-base ConC trimer immunized groups in guinea pigs

Group	Clade / Virus / Animal ID	Non-HIV SIVmac251	Non-HIV SVA-MLV	C ConC	C ConC_FPV2. N611Q	A BG505. N611Q	C MW965.26
2207: Protein-base ConC	2207-1	<20	<20	<20	<20	<20	<20
	2207-2	<20	<20	<20	<20	<20	<20
	2207-3	<20	<20	<20	<20	<20	<20
	2207-4	<20	<20	<20	<20	<20	<20
	2207-5	<20	<20	<20	<20	<20	<20
	2207-6	<20	<20	<20	<20	<20	<20
	2207-7	<20	<20	<20	<20	<20	<20
	2207-8	<20	<20	<20	<20	<20	<20
	2207-9	<20	<20	<20	<20	<20	<20
	2207-10	<20	<20	<20	<20	<20	<20
2208: Glycan-base ConC	2208-1	<20	<20	<20	<20	<20	<20
	2208-2	<20	<20	<20	<20	<20	<20
	2208-3	<20	<20	<20	<20	<20	<20
	2208-4	<20	<20	<20	<20	<20	<20
	2208-5	<20	<20	<20	<20	<20	90
	2208-6	<20	<20	<20	<20	<20	<20
	2208-7	<20	<20	<20	<20	<20	<20
	2208-8	<20	<20	<20	<20	<20	<20
	2208-9	<20	<20	<20	<20	<20	25
	2208-10	<20	<20	<20	<20	<20	<20

Table S4. Background-corrected ELISA data, related to Figures 6 and 7, and Figure S5.

			Mouse 341 (Protein-base BG505 immunized)										Mouse 342 (Glycan-base BG505 immunized)									
		Dilution factor	E01	E02	E03	E04	E05	E06	E07	E08	E09	E10	E11	E12	E13	E14	E15	E16	E17	E18	E19	E20
anti-Protein base BG505	Week 8	100	2.613	2.655	2.590	1.835	1.891	2.917	2.626	2.620	2.353	2.347	0.351	0.342	-0.005	0.004	0.013	0.25	0.68	0.28	-0.05	0.09
		500	2.546	1.672	2.517	2.284	1.681	2.913	2.434	2.238	2.496	2.158	-0.020	-0.052	-0.048	-0.059	-0.061	-0.06	-0.08	-0.07	-0.11	-0.07
		2,500	2.011	2.279	1.292	1.569	1.087	2.712	2.617	1.786	2.072	1.991	-0.052	-0.070	-0.078	-0.071	-0.078	-0.03	-0.12	-0.11	-0.12	-0.12
		12,500	1.203	1.652	0.574	0.313	0.579	2.107	1.729	0.723	0.964	1.087	-0.036	-0.074	-0.077	-0.081	-0.078	-0.06	-0.12	-0.13	-0.13	-0.13
		62,500	0.420	0.256	0.160	0.058	0.137	1.130	0.687	0.259	0.384	0.431	0.001	-0.077	-0.079	-0.079	-0.080	-0.07	-0.12	-0.13	-0.13	-0.13
		312,500	0.105	0.045	0.007	0.002	0.009	0.550	0.245	0.053	0.086	0.083	-0.044	-0.080	-0.082	-0.072	-0.083	-0.05	-0.12	-0.12	-0.13	-0.13
		1,562,500	-0.014	0.001	-0.009	-0.027	-0.020	0.352	0.072	-0.016	-0.002	0.004	-0.038	-0.062	-0.071	-0.077	-0.071	-0.01	-0.09	-0.12	-0.11	-0.12
anti-Glycan base BG505	Week 8	100	0.04	0.13	0.08	0.12	0.04	0.47	0.28	0.07	0.07	0.34	1.08	1.17	1.39	1.36	0.95	1.49	1.24	1.40	1.34	0.98
		500	-0.02	0.02	-0.02	0.00	-0.02	0.09	0.11	0.00	-0.01	0.14	0.76	0.68	0.58	0.77	1.14	0.78	0.86	1.05	0.86	0.80
		2,500	-0.03	-0.03	-0.03	-0.03	-0.03	0.02	0.06	-0.02	-0.02	0.08	0.43	0.24	0.19	0.30	0.59	0.27	0.39	0.43	0.34	0.30
		12,500	-0.04	-0.03	-0.04	-0.04	-0.04	0.02	-0.02	-0.03	-0.03	0.00	0.09	0.02	0.00	0.04	0.18	0.05	0.08	0.10	0.06	0.04
		62,500	-0.04	-0.03	-0.03	-0.03	-0.04	-0.02	-0.01	-0.02	-0.02	-0.02	-0.02	-0.04	-0.03	-0.03	0.01	-0.02	-0.01	0.00	-0.01	-0.01
		312,500	-0.04	-0.04	-0.04	-0.03	-0.04	-0.02	-0.02	-0.03	-0.02	-0.03	-0.05	-0.05	-0.05	-0.05	-0.04	-0.03	-0.03	-0.02	-0.02	-0.03
		1,562,500	-0.04	-0.04	-0.04	-0.04	-0.03	-0.02	-0.03	-0.03	-0.03	-0.03	-0.05	-0.05	-0.05	-0.04	-0.05	-0.03	-0.03	-0.03	-0.02	-0.03

			Mouse 343 (Protein-base ConC immunized)										Mouse 344 (Glycan-base ConC immunized)									
		Dilution factor	E21	E22	E23	E24	E25	E26	E27	E28	E29	E30	E31	E32	E33	E34	E35	E36	E37	E38	E39	E40
anti-Protein base ConC	Week 8	100	1.732	1.772	2.133	1.713	1.985	2.039	1.997	1.989	1.888	1.819	0.831	0.931	0.214	0.640	0.224	0.22	0.17	0.49	0.10	0.12
		500	1.910	1.647	2.150	1.895	2.048	2.065	1.535	2.018	1.956	1.595	0.677	0.288	0.009	0.253	0.003	0.01	0.03	0.11	-0.01	-0.02
		2,500	0.979	1.008	1.864	1.772	1.629	1.838	1.016	1.789	1.416	0.988	0.036	0.097	-0.001	0.077	-0.019	-0.04	-0.03	-0.03	-0.06	-0.06
		12,500	0.341	0.345	1.377	1.282	1.050	1.396	0.344	1.515	0.636	0.309	-0.022	0.001	-0.035	-0.017	-0.038	-0.03	-0.06	-0.06	-0.07	-0.07
		62,500	0.064	0.047	0.548	0.474	0.382	0.476	0.054	0.749	0.144	0.047	0.017	-0.031	-0.031	-0.031	-0.038	-0.05	-0.07	-0.07	-0.07	-0.07
		312,500	0.049	-0.015	0.111	0.095	0.063	0.138	-0.035	0.228	-0.008	-0.039	-0.039	-0.039	-0.040	-0.036	-0.043	-0.05	-0.07	-0.07	-0.07	-0.08
		1,562,500	0.001	0.000	0.006	0.002	-0.004	0.006	-0.051	0.058	-0.046	-0.054	-0.001	-0.032	-0.035	-0.039	-0.040	-0.06	-0.07	-0.07	-0.07	-0.07
anti-Glycan base ConC	Week 8	100	0.27	0.05	0.17	0.04	0.02	0.03	0.74	-0.03	-0.02	0.02	0.95	1.64	1.24	2.45	1.10	1.00	1.22	1.15	1.28	0.74
		500	0.07	-0.01	0.07	0.00	-0.01	-0.04	0.00	-0.05	-0.05	-0.04	0.54	1.17	0.87	1.75	0.63	0.76	1.02	0.87	0.80	0.45
		2,500	0.01	-0.01	0.01	-0.01	-0.01	-0.05	-0.04	-0.05	-0.05	-0.05	0.25	0.56	0.47	0.95	0.20	0.31	0.49	0.35	0.39	0.17
		12,500	-0.01	-0.01	-0.01	-0.01	-0.01	-0.05	-0.05	-0.05	-0.05	-0.05	0.07	0.17	0.15	0.33	0.04	0.10	0.15	0.09	0.12	0.03
		62,500	0.00	-0.01	-0.01	-0.01	-0.01	-0.05	-0.05	-0.05	-0.05	-0.05	0.01	0.04	0.04	0.08	0.00	0.02	0.03	0.02	0.03	0.00
		312,500	-0.01	-0.01	-0.01	-0.02	-0.01	-0.05	-0.05	-0.05	-0.05	-0.05	0.00	0.01	0.00	0.01	-0.01	0.00	0.00	-0.01	0.00	0.00
		1,562,500	-0.01	0.00	0.00	0.01	-0.01	-0.04	-0.05	-0.04	-0.04	-0.04	-0.01	-0.01	-0.01	0.00	-0.01	-0.01	-0.01	-0.01	-0.01	-0.01

			GP2108 (Protein-base BG505 immunized)										GP2109 (Glycan-base BG505 immunized)									
		Dilution factor	GP2108-1	GP2108-2	GP2108-3	GP2108-4	GP2108-5	GP2108-6	GP2108-7	GP2108-8	GP2108-9	GP2108-10	GP2109-1	GP2109-2	GP2109-3	GP2109-4	GP2109-5	GP2109-6	GP2109-7	GP2109-8	GP2109-9	GP2109-10
anti-Protein base BG505	Week 6	1,000	3.295	3.193	1.464	2.021	1.799	3.436	3.406	3.435	3.424	3.359	0.515	0.518	-0.054	0.991	0.033	0.32	0.99	0.49	0.89	0.39
		5,000	3.165	2.822	0.270	0.993	0.523	3.443	3.409	3.352	3.387	3.335	0.142	-0.007	-0.128	0.231	-0.102	0.09	0.24	0.29	0.36	0.08
		25,000	1.988	1.057	0.275	0.311	0.175	2.977	2.413	2.749	2.767	2.238	0.141	-0.103	-0.138	-0.060	-0.117	0.04	0.03	0.01	0.06	0.02
		125,000	0.618	0.106	0.041	-0.012	0.018	1.393	0.789	1.070	1.342	0.699	0.041	-0.128	-0.145	-0.113	-0.141	0.00	-0.03	-0.04	-0.03	-0.02
		625,000	0.144	-0.024	-0.043	-0.039	-0.035	0.516	0.193	0.274	0.422	0.188	-0.137	-0.123	-0.146	-0.136	-0.143	-0.01	0.09	-0.04	-0.05	-0.04
		3,125,000	0.052	-0.043	-0.046	-0.050	-0.047	0.200	0.066	0.075	0.123	0.041	-0.135	-0.112	-0.146	-0.144	-0.139	0.00	-0.04	-0.05	-0.05	-0.05
		15,625,000	0.048	-0.036	-0.033	-0.047	-0.040	0.214	-0.013	0.017	0.008	-0.014	-0.106	-0.049	-0.113	-0.135	-0.129	0.06	-0.02	-0.03	-0.04	-0.03
anti-Glycan base BG505	Week 6	1,000	0.08	0.18	0.30	0.35	0.48	0.43	0.09	0.37	0.28	0.67	2.58	3.04	1.28	2.51	2.54	2.53	3.26	2.59	3.12	3.26
		5,000	-0.07	-0.04	-0.02	-0.04	0.02	0.10	-0.01	0.09	0.07	0.29	1.75	2.83	0.71	2.29	1.23	1.73	2.80	2.43	2.89	2.33
		25,000	-0.07	-0.07	-0.05	-0.03	-0.02	-0.01	-0.04	-0.02	-0.02	0.01	0.55	1.30	0.30	0.83	0.32	0.55	0.98	1.08	1.14	0.64
		125,000	-0.07	-0.06	-0.06	-0.07	-0.04	-0.03	-0.04	-0.04	-0.03	0.00	0.09	0.29	0.02	0.21	0.08	0.08	0.36	0.31	0.30	0.14
		625,000	-0.07	-0.07	-0.07	-0.05	-0.04	-0.04	-0.04	-0.03	-0.03	0.00	-0.03	0.05	-0.04	-0.02	-0.02	-0.03	0.02	0.06	0.00	0.03
		3,125,000	-0.07	-0.07	-0.06	-0.04	0.00	-0.04	-0.04	-0.04	-0.04	-0.01	-0.06	-0.03	-0.06	-0.02	-0.03	-0.06	-0.05	-0.03	-0.01	-0.03
		15,625,000	-0.06	-0.06	-0.05	-0.03	-0.03	-0.03	-0.04	-0.03	-0.02	0.01	-0.05	-0.03	-0.02	-0.02	0.00	-0.06	-0.05	-0.03	0.01	0.00

			GP2207 (Protein-base ConC immunized)										GP2208 (Glycan-base ConC immunized)									
		Dilution factor	GP2207-1	GP2207-2	GP2207-3	GP2207-4	GP2207-5	GP2207-6	GP2207-7	GP2207-8	GP2207-9	GP2207-10	GP2208-1	GP2208-2	GP2208-3	GP2208-4	GP2208-5	GP2208-6	GP2208-7	GP2208-8	GP2208-9	GP2208-10
anti-Protein base ConC	Week 6	1,000	2.021	2.758	2.634	2.465	2.754	2.513	2.753	2.974	3.089	3.160	-0.015	-0.132	-0.082	-0.120	-0.069	-0.030	-0.056	-0.076	0.022	-0.087
		5,000	1.944	2.355	1.503	1.582	2.336	2.358	2.406	3.067	2.302	2.767	-0.103	-0.158	-0.123	-0.132	-0.118	-0.097	-0.100	-0.087	-0.093	-0.108
		25,000	1.073	1.744	0.839	0.486	1.561	1.000	1.501	2.683	0.943	1.260	-0.144	-0.166	-0.123	-0.163	-0.164	-0.108	-0.074	-0.085	-0.093	-0.066
		125,000	0.291	0.547	0.166	0.047	0.480	0.290	0.429	1.303	0.246	0.442	-0.134	-0.148	-0.141	-0.169	-0.143	-0.122	-0.127	-0.125	-0.119	-0.126
		625,000	0.050	0.117	-0.019	-0.041	0.065	0.079	0.106	0.376	0.050	0.104	-0.143	-0.160	-0.141	-0.144	-0.129	-0.123	-0.125	-0.122	-0.122	-0.125
		3,125,000	-0.020	-0.027	-0.057	-0.062	-0.003	0.030	0.057	0.110	0.007	0.020	-0.156	-0.158	-0.159	-0.170	-0.156	-0.121	-0.129	-0.127	-0.131	-0.131
		15,625,000	-0.037	-0.054	-0.066	-0.067	-0.064	0.017	0.001	0.016	-0.004	-0.002	-0.150	-0.167	-0.152	-0.132	-0.158	-0.118	-0.125	-0.113	-0.126	-0.129
anti-Glycan base ConC	Week 6	1,000	0.019	-0.044	-0.029	-0.044	-0.041	0.027	0.040	0.008	0.073	0.021	2.285	1.935	2.114	1.574	1.546	1.624	1.692	1.379	1.974	1.137
		5,000	-0.055	-0.070	-0.067	-0.064	-0.067	-0.003	0.005	-0.008	0.007	-0.004	1.096	1.089	1.233	0.560	0.898	0.629	0.866	0.402	0.908	0.642
		25,000	-0.069	-0.071	-0.069	-0.065	-0.052	-0.008	-0.008	-0.008	-0.007	-0.007	0.260	0.363	0.411	-0.069	0.271	0.158	0.238	0.074	0.241	0.139
		125,000	-0.060	-0.072	-0.070	-0.070	-0.070	-0.008	-0.009	-0.005	-0.007	-0.007	-0.088	-0.087	-0.074	-0.122	-0.093	-0.070	0.000	-0.084	-0.035	-0.047
		625,000	-0.069	-0.069	-0.059	-0.063	-0.068	0.005	0.000	0.030	-0.006	-0.005	-0.129	-0.105	-0.106	-0.145	-0.125	-0.116	-0.098	-0.119	-0.102	-0.120
		3,125,000	-0.072	-0.063	-0.063	-0.064	-0.072	-0.010	-0.002	-0.009	-0.007	-0.007	-0.165	-0.139	-0.156	-0.169	-0.163	-0.118	-0.125	-0.129	-0.124	-0.126
		15,625,000	-0.067	-0.070	-0.072	-0.038	-0.069	-0.007	-0.001	-0.008	-0.003	-0.006	-0.166	-0.159	-0.165	-0.167	-0.159	-0.110	-0.121	-0.130	-0.126	-0.127

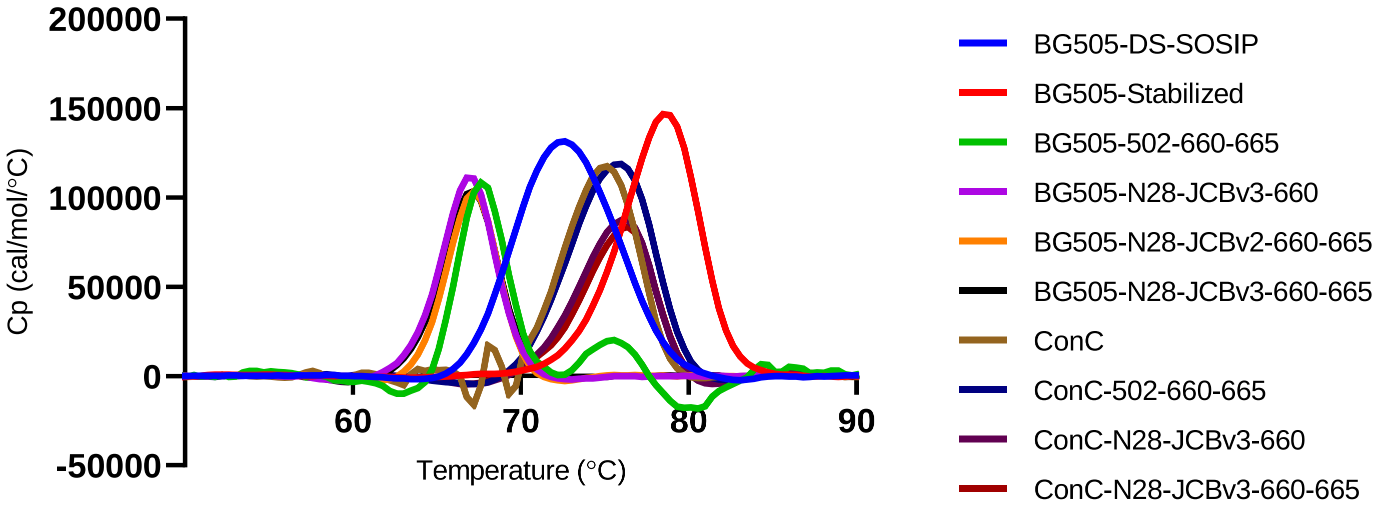

Constructs	Melting Temperature (°C)
BG505-DS-SOSIP	72.6
BG505-Stabilized	78.5
BG505-502-660-665	67.7
BG505-N28-JCBv3-660	66.9
BG505-N28-JCBv2-660-665	67.3
BG505-N28-JCBv3-660-665	67.3
ConC	75.2
ConC-502-660-665	76.0
ConC-N28-JCBv3-660	76.0
ConC-N28-JCBv3-660-665	76.5

Figure S1. Differential Scanning Calorimetry for top BG505 and ConC base-covered variants, related to Figure 2. DSC scans of glycan-base candidates for BG505 and ConC as compared to the protein-base templates, with calculated melting temperature (T_m) for each construct.

A

B

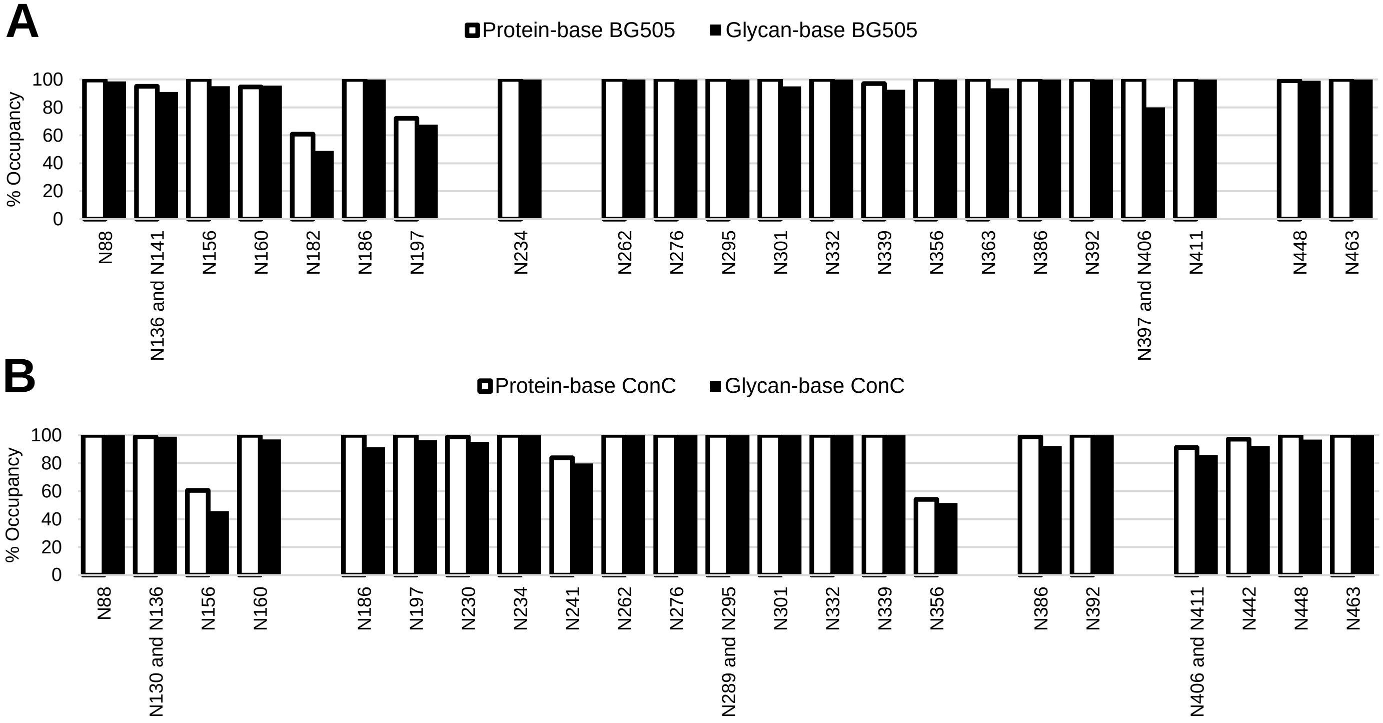

C

BG505 (% Glycosylation)		
	Protein-base	Glycan-base
N88	99.5	98.6
N136/141	95.1	91.0
N156	100	95.2
N160	94.6	95.6
N182	60.8	48.9
N186	100	100
N197	72.2	67.7
N234	100	100
N262	100	100
N276	100	100
N295	100	100
N301	100	95
N332	100	100
N339	97	92.6
N356	100	100
N363	100	93.7
N386	100	100
N392	100	100
N397/406	100	80
N411	100	100
N448	99.1	99
N463	100	100
N502	N/A	100
N508	N/A	0
N512	N/A	100

ConC (% Glycosylation)		
	Protein-base	Glycan-base
N88	100	100
N130/ N136	99	99
N156	60.6	45.7
N160	100	97.1
N186	100	91.4
N197	100	96.5
N230	99	95.3
N234	100	100
N241	83.9	79.9
N262	100	100
N276	100	100
N289/295	100	100
N301	100	100
N332	100	100
N339	100	100
N356	54.2	51.6
N386	98.9	92.4
N392	100	100
N406/411	91.3	86
N442	97.2	92.4
N448	100	96.9
N463	100	100
N502	N/A	100
N508	N/A	20
N512	N/A	100

Figure S2. Glycan analysis of gp120 of protein-base and glycan-base trimers, related to Figure 3. (A) Percent glycan occupancy at each glycosylation site in gp120 of either protein-base or glycan base BG505 or (B) ConC. (C) Occcupancy values at each site.

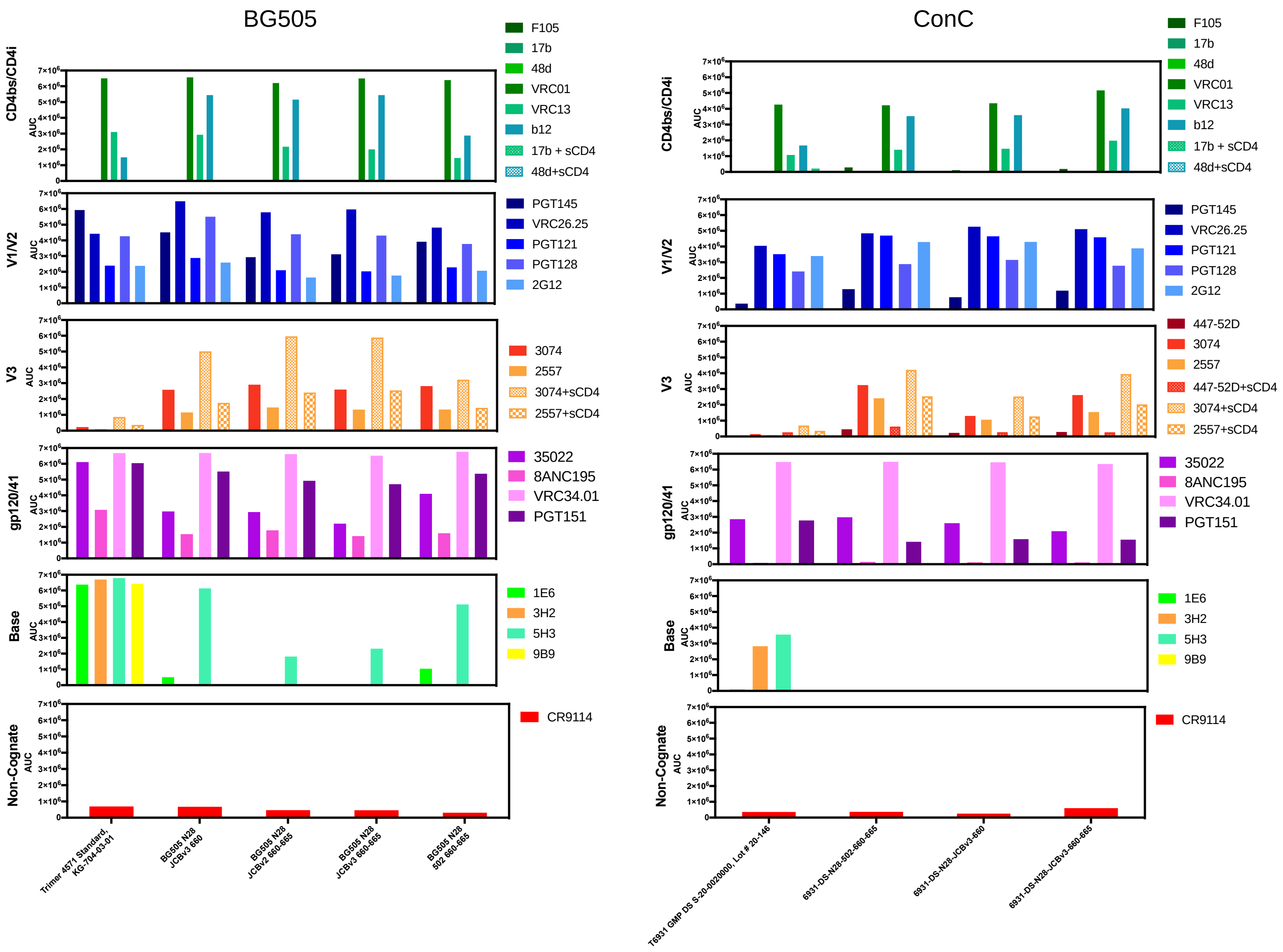

Figure S3. MSD for top BG505 and ConC base-covered variants, related to Figure 3. MSD antigenicity analysis of glycan-base candidates for BG505 and ConC. In both cases, glycan-base constructs show similar binding to antibody panel as the protein-base counterpart, with the exception of slightly high V3 reactivity in most cases.

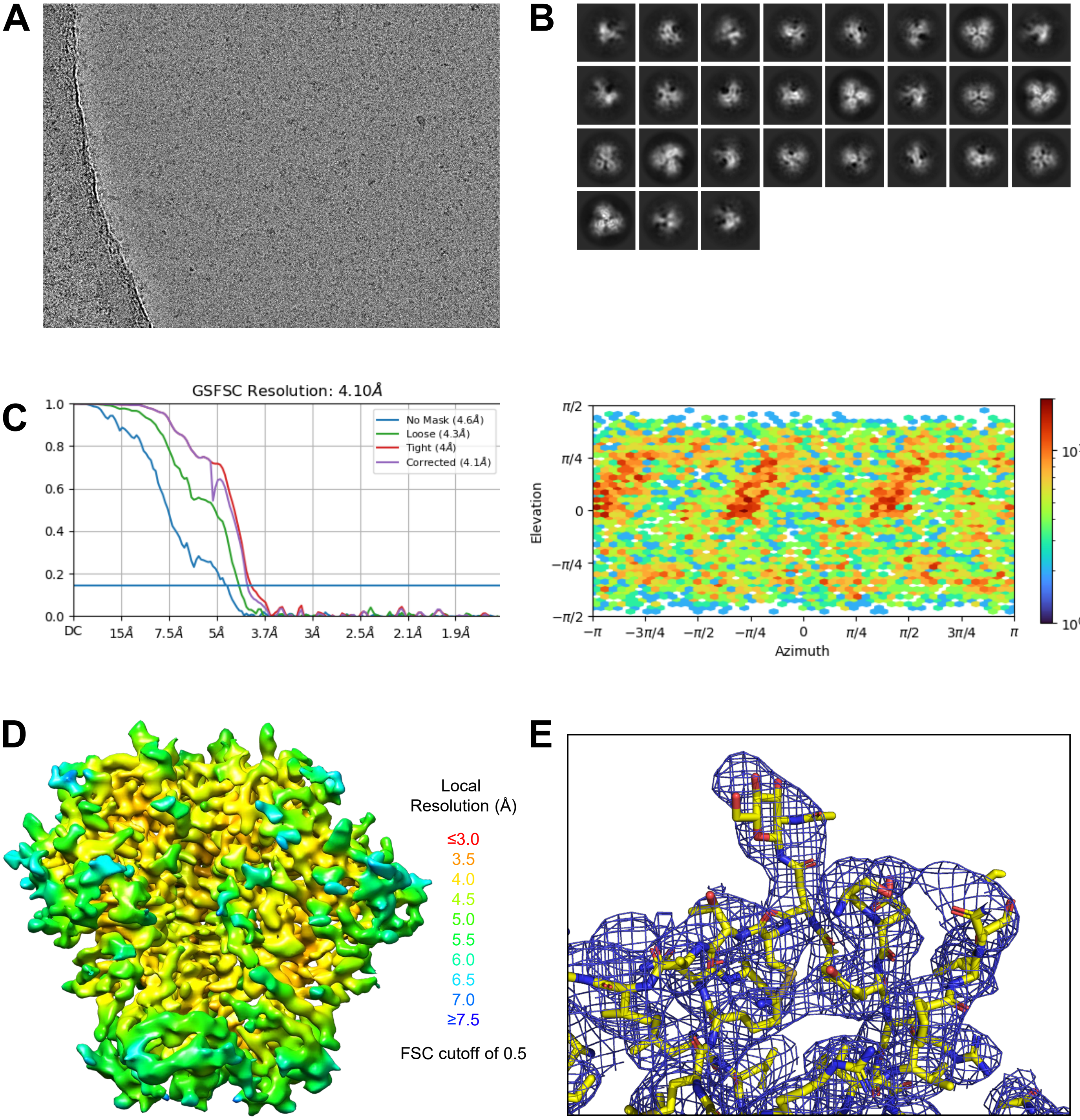

Figure S4. Cryo-EM details of Glycan-base ConC, related to Figure 4. (A) Representative micrograph. (B) Representative 2D class averages. (C) The gold-standard Fourier shell correlation resulted in a resolution of 3.39 Å for the overall map using non-uniform refinement with C3 symmetry (left panel); the orientations of all particles used in the final refinement are shown as a heatmap (right panel). (D) Local resolution of the final reconstruction, shown by color coding from red to blue for high to low resolution. (E) Representative map after sharping and improvement in Resolve. Map is contoured at 2σ.

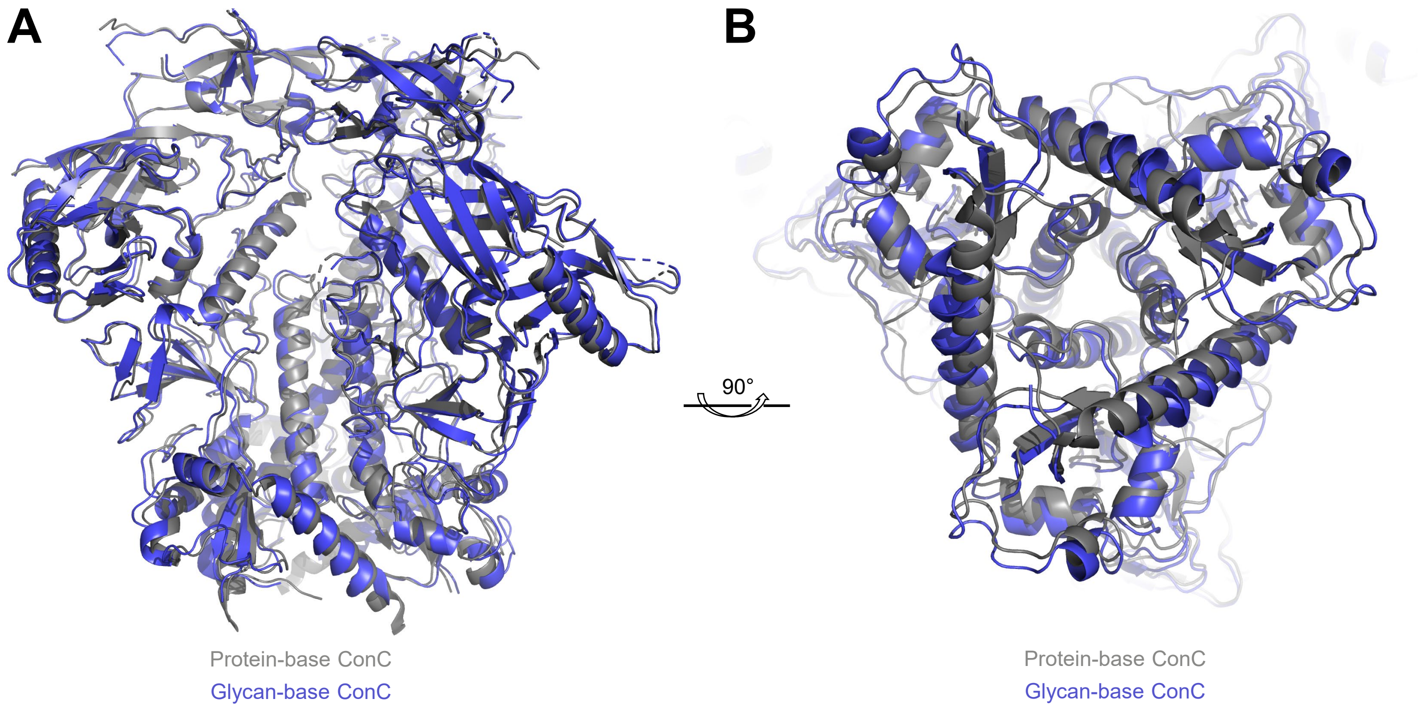

Figure S5. Superposition of protein-base ConC structure onto glycan-base ConC, related to Figure 4. (A) Structural superposition of published protein-base ConC structure (PDB 6CK9) in grey with the glycan-base ConC structure in blue. (B) Bottom view of the superposition showing the movement at the base of the trimer.

A

Group	Animal ID	Anti-protein-base BG505 ELISA endpoint titer (Week 8)	Anti-glycan-base BG505 ELISA endpoint titer (Week 8)	Cross-reactivity (%)
341: Protein-base BG505	E01	312500	300	0.10
	E02	187500	300	0.16
	E03	62500	100	0.16
	E04	37500	100	0.27
	E05	62500	100	0.16
	E06	4687500	300	0.01
	E07	937500	300	0.03
	E08	187500	300	0.16
	E09	187500	100	0.05
	E10	187500	100	0.05
342: Glycan-base BG505	E11	100	7500	1.33
	E12	100	7500	1.33
	E13	100	2500	4.00
	E14	100	7500	1.33
	E15	100	12500	0.80
	E16	300	7500	4.00
	E17	500	7500	6.67
	E18	100	12500	0.80
	E19	100	7500	1.33
	E20	500	7500	6.67

Group	Animal ID	Anti-Protein-Base ConC ELISA Endpoint Titer (Week 8)	Anti-Glycan-Base ConC ELISA Endpoint Titer (Week 8)	% Cross-Reactivity
343: Protein-base ConC	E21	37500	1500	4.00
	E22	37500	1500	4.00
	E23	312500	300	0.10
	E24	187500	1500	0.80
	E25	187500	300	0.16
	E26	312500	300	0.10
	E27	37500	100	0.27
	E28	937500	500	0.05
	E29	62500	100	0.16
	E30	37500	100	0.27
344: Glycan-base ConC	E31	300	7500	4.00
	E32	100	12500	0.80
	E33	100	12500	0.80
	E34	100	37500	0.27
	E35	100	7500	1.33
	E36	100	7500	1.33
	E37	300	12500	2.40
	E38	100	7500	1.33
	E39	100	12500	0.80
	E40	100	2500	4.00

B

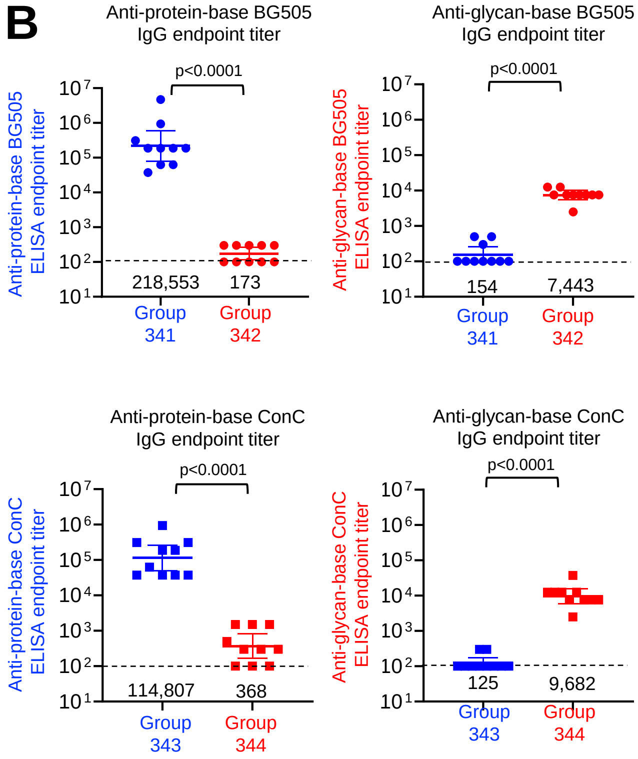

C

Group	Clade / Virus / Animal ID	Non-HIV SIVmac251	A BG505.W6M,C2	C ConC	C MW965.26
341: Protein-base BG505	E01	<50	<50	<50	<50
	E02	<50	<50	<50	<50
	E03	<50	<50	<50	<50
	E04	<50	<50	<50	<50
	E05	<50	<50	<50	<50
	E06	<50	<50	<50	<50
	E07	<50	<50	<50	<50
	E08	<50	<50	<50	<50
	E09	<50	<50	<50	<50
	E10	<50	<50	<50	<50
342: Glycan-base BG505	E11	<50	<50	<50	<50
	E12	<50	<50	<50	<50
	E13	<50	<50	<50	<50
	E14	<50	<50	<50	<50
	E15	<50	<50	<50	<50
	E16	<50	<50	<50	<50
	E17	<50	<50	<50	<50
	E18	<50	<50	<50	<50
	E19	<50	<50	<50	<50
	E20	<50	<50	<50	<50
343: Protein-base ConC	E21	<50	<50	<50	<50
	E22	<50	<50	<50	<50
	E23	<50	<50	<50	<50
	E24	<50	<50	<50	<50
	E25	<50	<50	<50	<50
	E26	<50	<50	<50	<50
	E27	<50	<50	<50	<50
	E28	<50	<50	<50	<50
	E29	<50	<50	<50	<50
	E30	<50	<50	<50	<50
344: Glycan-base ConC	E31	<50	<50	<50	<50
	E32	<50	<50	<50	<50
	E33	<50	<50	<50	<50
	E34	<50	<50	<50	<50
	E35	<50	<50	<50	257
	E36	<50	<50	<50	<50
	E37	<50	<50	<50	<50
	E38	<50	<50	<50	<50
	E39	<50	<50	<50	<50
	E40	<50	<50	<50	<50

Figure S6. ELISA endpoint titers and serum neutralization (ID_{50}) titers elicited at Week 8 for BG505 or ConC trimer-immunized mice, related to Figure 5. (A) Week 8 ELISA endpoint titers using a starting dilution of 1:100 and cross-reactivity (%) of responses are listed for each animal (B) Group comparison shown for week 8 ELISA endpoint titers against protein-base and glycan-base variants of BG505 trimer (left) and ConC trimer (right) (C) Week 8 neutralization (ID_{50}) titers against wildtype BG505, ConC, MW965.26, and SIVmac251 strains.
